# Computational Mass Spectrometry Imaging in the Era of AI: Focus Review

**DOI:** 10.1021/acs.chemrev.6c00098

**Published:** 2026-08-05

**Authors:** Timothy J. Trinklein, Mithunjha Anandakumar, Hsi-Chun Chao, Marisa Asadian, Dharmeshkumar Parmar, Jonathan V. Sweedler, Fan Lam

**Affiliations:** † Department of Chemistry, 14589 University of Illinois Urbana−Champaign, Urbana, Illinois 61801, United States; ‡ Beckman Institute for Advanced Science and Technology, 14589 University of Illinois Urbana−Champaign, Urbana, Illinois 61801, United States; § Department of Bioengineering, 14589 University of Illinois Urbana−Champaign, Urbana, Illinois 61801, United States; ∥ Neuroscience Program, 14589 University of Illinois Urbana−Champaign, Urbana, Illinois 61801, United States; ⊥ Cancer Center at Illinois, 14589 University of Illinois Urbana−Champaign, Urbana, Illinois 61801, United States

## Abstract

Mass spectrometry imaging (MSI) is a tool-of-choice for mapping and understanding the spatial organization of biomolecules, including small metabolites, lipids, peptides, and many others. As the MSI instrument and spatial biology inquiries evolve, researchers and practitioners are constrained by the inherent trade-offs in spatial resolution, chemical detail, and acquisition time. Here, we review how the rapidly growing interplay between MSI and machine learning/artificial intelligence (ML/AI)-powered computational approaches is addressing these issues. We begin by highlighting key steps in MSI experiments and summarizing major ML/AI paradigms in the context of MSI data, providing a foundation to review how ML/AI impact each step in the MSI workflow, starting with methods to accelerate data acquisition. We then discuss emerging applications of dimensionality reduction, segmentation, and various supervised/unsupervised learning approaches to extract useful chemical insights from high-dimensional MSI data. Approaches to leverage multimodal imaging to guide the acquisition process or provide a more informative integrated analysis are discussed. We conclude with a forward-looking discussion on the state of computation and MSI, spanning ML-enabled instrumentation, scaling measurements to 3D and large cohorts, and the integration of MSI with other spatial omic data.

## Graphical abstract

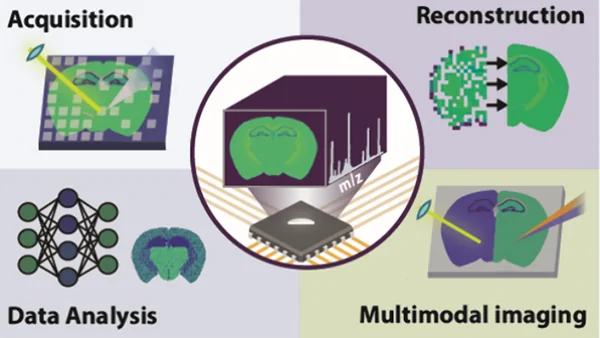

## 1 Introduction

Understanding the spatial distribution of biomolecules is critical to decode biological complexity, discover disease mechanisms, and identify diagnostic markers and treatment targets. As rich spatial heterogeneities in the molecular portraits across organs, tissues, cells, and organelles, under physiological and pathological conditions, continue to be unveiled using tools such as spatial transcriptomics, there is an ever-increasing interest in resolving similar and distinct patterns at different scales for small metabolites, neurotransmitters, lipids, peptides, proteins, and carbohydrates. With the unmatched combination of chemical specificity and sensitivity, mass spectrometry imaging (MSI) has emerged as the tool-of-choice for spatially resolved, multiplexed biochemical analysis.^1–3^ MSI enables label-free mapping of hundreds and potentially thousands of biomolecules directly from tissue, revealing patterns and heterogeneity that are otherwise inaccessible.

Since its first applications to large intact biomolecules in the late 1990s,^4–6^ MSI has rapidly evolved from an academic exercise into a leading tool in spatial biology.^1–3^ MSI provides exceptional chemical detail, uncovering complex heterogeneities in cells, tissues, and organs, driving its translation to biomedicine. Recent developments in instrumentation, sample preparation, measurement strategies, and data analysis tools have dramatically improved the spatial resolution, molecular coverage, and chemical specificity achievable with MSI. Today, standard MSI instrumentation is capable of detecting thousands of masses resulting from hundreds of unique metabolites, lipids, and other biomolecules within a single imaging run. With the increasing ambition to make single cell analysis routine, and push toward imaging entire organs, the scale and complexity of MSI data and experiments has exploded.

With advances in instrumentation and a growing wealth of complex, high-dimensional data, efficient acquisition and robust analysis are becoming increasingly important, giving rise to a few key challenges and opportunities. *First*, there are inherent trade-offs in the achievable resolution (pixel size), chemical detail, tissue coverage, and data acquisition time. For instance, compared to the more commonly used 50 μm or larger pixel sizes in most MSI studies, imaging at a 10 μm pixel size or below reveals richer tissue heterogeneities^7,8^ but takes 25× longer and reduces analyte detectability as there is 25× less material per pixel. As a result, these experiments are typically limited to small, targeted regions (<2 mm^2^).^9^ This calls for significantly improved speeds for high-resolution acquisitions. *Second*, despite unmatched chemical detail, the spatial resolution of MSI still trails far behind other ex vivo imaging modalities, such as optical microscopies, which are routinely performed at submicron resolutions. To circumvent this limit, physical sample isolation has been used to achieve single-cell and even single-organelle MS measurement.^10–12^ The ability to effectively bridge these different scales and push past the practical MSI resolution limits will not only advance the technology but also reveal new levels of biochemical detail. *Third*, the size and complexity of MSI data sets are growing exponentially, as illustrated in Figure 1a. It is now not uncommon to generate data sets with millions of spectra, each containing thousands of mass-to-charge (*m*/*z*) values when using high-mass-resolution instrumentation. Moreover, information-rich, multidimensional instruments, such as MSI with ion mobility, are maturing rapidly, generating enormous multidimensional data. The resulting data sets can easily contain tens of billions of intensity values.^13,14^ Additionally, molecular identification and annotation for MSI have yet to achieve the precision and reliability of conventional chromatography-coupled MS analyses such as LCMS. These challenges underscore the need for more advanced infrastructure and informatics pipelines to manage, analyze, visualize, and interpret MSI data efficiently, especially as practitioners push MSI toward answering new biological questions and into clinical settings.

**Figure 1:**
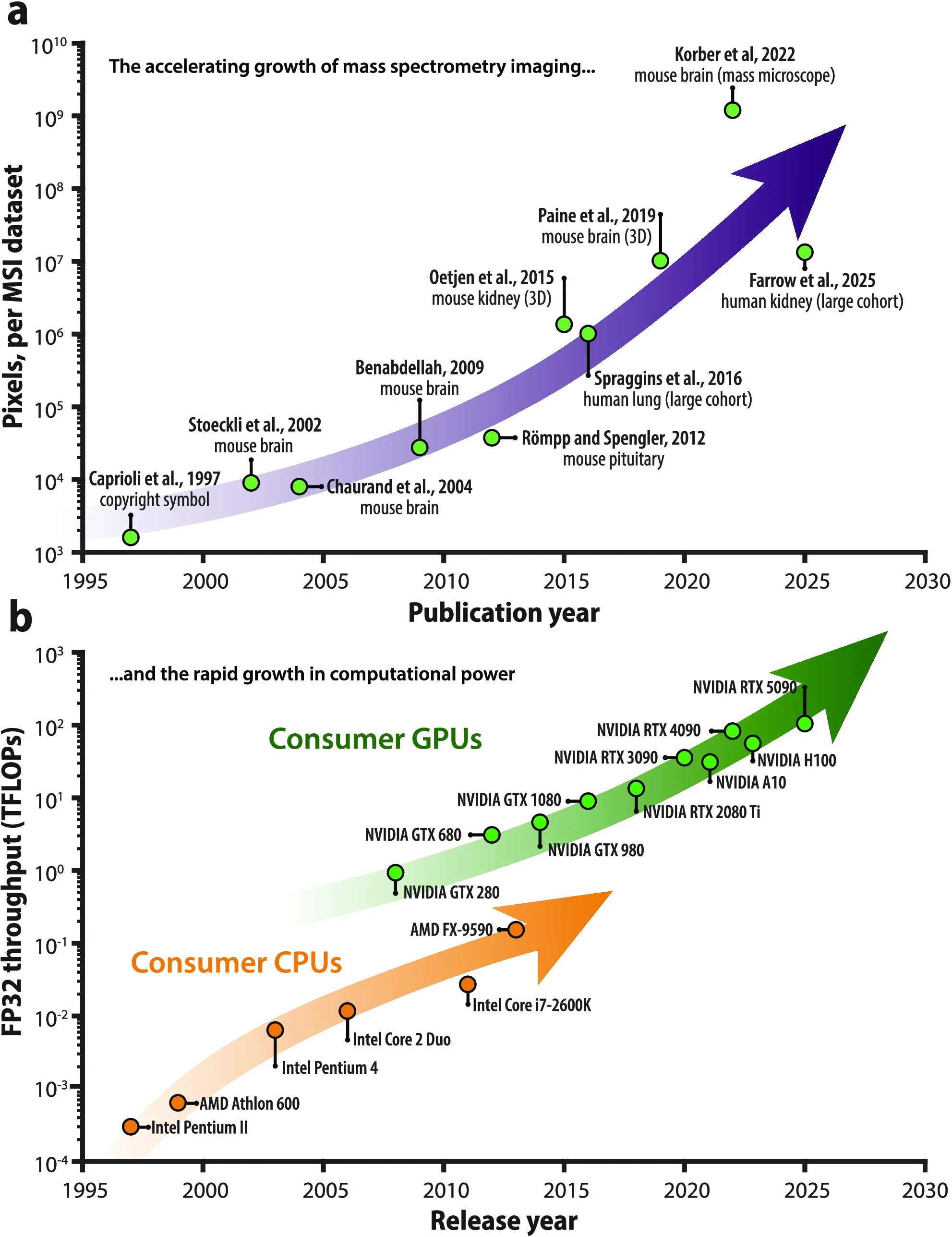
(a) Exponential growth in MSI data size and scope, as illustrated by the total number of pixels in an experiment for select MSI studies from 1995–present. (b) Likewise, computing power has grown at an explosive rate, e.g., in terms of TFLOPs, for consumer-level CPUs and GPUs, over the same period.

In concert with the development of MSI, artificial intelligence (AI) techniques and in particular machine learning (ML) have been developing at an explosive pace in recent years. As a broad discipline, recent exciting advances in AI and its many applications have been mostly driven by deep learning (DL), a collection of approaches that use multilayer artificial neural networks to model and derive domain-specific, rich features from large volumes of complex, high-dimensional data.^15^ The impressive performance of DL and its rapid adoption have been largely enabled by massively parallel computing offered by graphics processing units (GPUs), large public data sets, and algorithmic advances. Since the release of GPUs for scientific computing, the amount of computational power available for AI research and application surged and continues to grow exponentially (Figure 1b), transforming biomedical and biological imaging and many other fields.

These data-driven approaches and increasingly available high-performance computing resources present unique opportunities to address the throughput, resolution, dimensionality and analysis challenges associated with modern MSI instrumentation and applications. Over the past decade, we have seen a rapidly increasing volume of ML/AI-powered computational methods developed and applied to MSI, accelerating data acquisition,^16,17^ enhancing spatial and mass resolution,^18,19^ facilitating intuitive visualization,^20,21^ extracting biologically relevant information,^22,23^ and integrating MSI with other imaging modalities.^24,25^ The ever-increasing scale and complexity of MSI data being generated routinely will benefit tremendously from more automatic, high-throughput and reproducible analysis offered by AI tools. Further, the data-driven nature and unmatched power of AI for discovering patterns in large data sets beyond human comprehension lends itself well to mining high-dimensional and complex MSI data, either separately or jointly with data from other imaging modalities.^14,26^ As the synergistic use of AI and MSI is expected to expand significantly in the coming years, potentially transforming how MSI data are acquired and analyzed, a review of recent developments and applications of AI/ML methods for different steps in the MSI workflow as well as identification of future opportunities should be valuable for researchers and practitioners who are interested in pursuing research in relevant areas.

This review focuses on the interplay between MSI, ML/AI, and high-performance computing. We begin by outlining the MSI paradigm, discussing key steps in all MSI acquisitions which are agnostic to the specific instrument and data characteristics, as well as major learning paradigms applicable to MSI. We then discuss how cutting-edge computational strategies are being applied to address key challenges in all aspects of MSI, beyond just data processing. Another key distinction of our review is its focus on state-of-the-art AI-powered methods, particularly those using deep learning techniques in the past 10 years (though select papers from earlier years are included for background and completeness). With our emphasis on how ML/AI impacts the *imaging process* (i.e., acquisition, reconstruction, and data analysis), the issue of molecular annotation in MSI is not treated in depth, although we acknowledge that this is an important area.^27–29^ Special emphasis is given to the multidimensional nature and unique spatial-spectral characteristics of MSI data, which we hope may inspire new perspectives and approaches in future research. We conclude with a forward-looking discussion on how AI approaches can be integrated into new MSI instrumentation and define the next generation of multiomic imaging.

## 2 The MSI Paradigm

We set up our review via a computational lens by first summarizing key characteristics of MSI data, with emphasis on tissue sampling, acquisition, and data analysis, illustrated in Figure 2. This is intended to help readers build the connection to AI approaches and better appreciate their impacts on different aspects of MSI. Given the computational focus of our review, steps related to sample preparation are not covered, and we refer the reader to prior excellent reviews covering this topic.^30–34^

**Figure 2:**
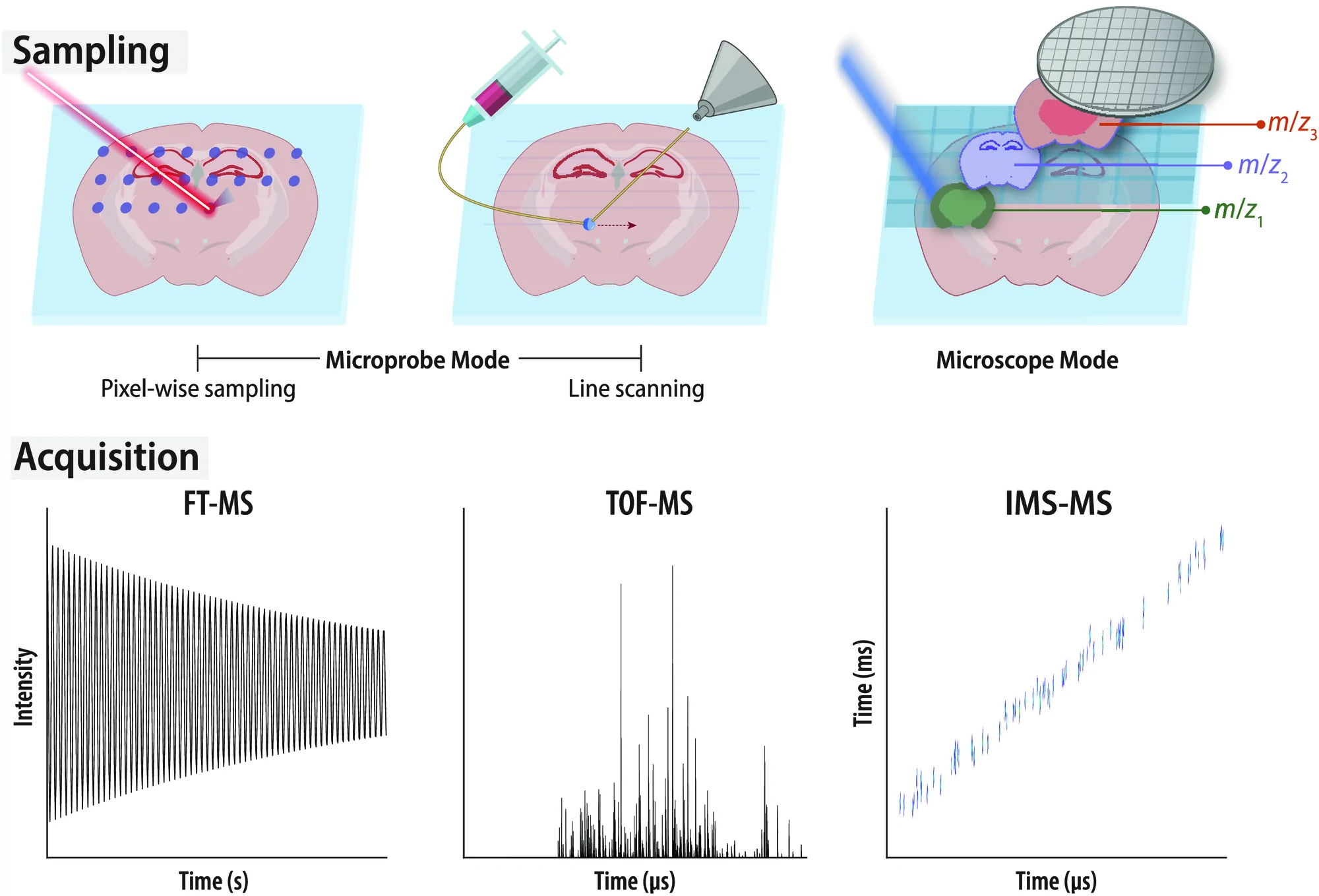
(Top) Methods for sampling data from a tissue section to the mass spectrometer. (Bottom) In Fourier transform MS (FT-MS), data are acquired as a time-domain signal (seconds long), which is then transformed to a frequency domain signal and converted to *m*/*z* using the instrument calibration. In TOF-MS, a time-domain signal (μs long) is directly converted to *m*/*z* using the instrument calibration. In ion mobility-MS, each pixel is obtained as a 2D array (mobility × *m*/*z*), generating a 4D data cube per sample.

### 2.1 Sampling

We define the sampling process as the manner in which mass spectra are obtained from spatially localized regions of the sample. Broadly, there are two modes for obtaining spatially resolved mass spectrometry data: microprobe mode and microscope mode. These two modes of operation are analogous to raster scanning and widefield modes in optical microscopy.

In microprobe mode (Figure 2 **),** a sampling and/or ionization probe is rastered across the sample. The microprobe may be the laser in matrix-assisted laser desorption ionization (MALDI),^34^ the ion beam in secondary ion MS (SIMS),^35,36^ the electrospray in desorption electrospray ionization (DESI),^37^ or the liquid junction in nanospray desorption electrospray ionization (nano-DESI), and this is the mode of operation for the overwhelming majority of MSI experiments. Within microprobe mode, sampling may be done pixel-wise or by line-scanning. In pixel-wise operation, the microprobe or an XY stage is moved in discrete steps, and one or more spectra are obtained and averaged at each location. At each step, the microprobe ionizes an area of tissue, one or more spectra are obtained, and the stage is moved again. This is the typical mode used in MALDI,^34^ and given the predominance of MALDI, it is the most common mode of operation.

In some cases, sampling may be obtained by line scanning. In this mode of operation, the probe is continuously moved in a line across the sample. In this case, the resulting pixel size is determined by a combination of the stage/probe velocity and the acquisition speed of the mass spectrometer. This is the standard operating mode for DESI^37^ and nano-DESI MSI,^38^ although it has also been applied for high-speed MALDI imaging.^39^ While the distinction between pixel-wise and line scanning-based sampling may seem minor, it can have drastic consequences in the design and application of computational solutions. One example we will explore later is the design of sparse sampling – in which a subset of the grids on the tissue is sampled and reconstructed in order to accelerate acquisition time.

Finally, in stark contrast to a pixel-wise or line scan “microprobe mode”, MSI data can be obtained in a “microscope mode”.^3,40^ In this case, an ionization source samples from a relatively large area of the sample at once and the ions are stigmatically transferred to a position-sensitive detector (Figure 2). This technique was employed in some of the earliest examples of MSI using SIMS with phosphor detector screens,^41^ and is seeing a renewed interest due to the development of high-speed, position-sensitive electronic detectors.^40,42^ Microscope mode has the potential to drastically enhance the throughput of MSI by decoupling spatial resolution from the ionization probe size. However, the technique offers poorer mass resolution (at least currently) and is only compatible with stigmatic mass analyzers such as time-of-flight (TOF) systems, limiting the extent of ion manipulations that can be performed. Therefore, it is not yet commercially available or widely used, but the unique data acquisition method and characteristics have interesting implications for computational strategies and deserve mention.

### 2.2 Acquisition and Data Characteristics

At each sampled pixel in a MSI experiment, a mass spectrum is obtained – we define this step as “*acquisition*”. The acquisition step determines the resulting data size and processing steps that can be applied. We consider three examples that are representative of the vast majority of MSI experiments (Figure 2): Fourier transform (FT)-MS, TOF-MS, and ion mobility-MS (IMS-MS). With FT-MS, a time-domain transient with harmonics at various frequencies is acquired. This transient can be converted into a spectral domain representation via a Fourier transform, and then the frequencies are converted to *m*/*z* values based on the instrument’s calibration. Typical transients may be on the order of 1–2 s but may exceed 10 s in some imaging experiments^43^ to resolve isobars and isotopic fine structure, making the transient length a major time-limiting factor in FT-MS-based MSI experiments.

In TOF-MS, a distribution of ion flight times from the ion source to detector is obtained on the order of microseconds. The time axis of this spectrum is then converted to *m*/*z* using the instrument’s mass calibration. Because each spectrum is acquired so quickly, TOF-MSI acquisition is much faster compared to FT-MSI given the same sampling area and number of pixels.

In ion mobility mass spectrometry (IM-MS), an ion mobility^44–46^ separation is nested between the ion source and the mass analyzer, most commonly a TOF-MS. Mass spectra are obtained during the ion mobility separation, generating a 2D data array for every pixel, with one axis denoting *m*/*z* and the other ion mobility. Although the resulting data are higher dimensional, the MS and IM dimensions are not completely independent and show strong correlation, presenting significant room for potential accelerated acquisition.

These different sampling and acquisition modes can all be treated as ways to generate a multidimensional spatial-spectral function of interest, with the spectral dimension containing rich molecular information. This function can be denoted as a multidimensional matrix/tensor, e.g., $\mathrm{X̃}\in R^{m\times n\times Q}$ (2D imaging), $\mathrm{X̃}\in R^{m\times n\times G\times Q}$ (2D MSI with IM), or $\mathrm{X̃}\in R^{m\times n\times o\times Q}$ (volumetric/3D imaging), where *m*, *n* and *o* denote the number of pixels for each spatial dimension, *G* an ion mobility dimension, and *Q* the *m*/*z* dimension (after peak picking). The tensor $\mathrm{X̃}\in R^{m\times n\times Q}$ can also be reshaped to a matrix $X\in R^{P\times Q}$ , where *P* represents the total number of pixels. This multidimensional data perspective provides an angle to appreciate the roles and impact of machine learning and AI tools in MSI.

## 3 Learning Paradigms

In this section, we provide a brief overview of the major paradigms in machine learning (ML): **supervised learning, unsupervised learning,** and **reinforcement learning**, in the context of MSI. The goal here is to connect ML concepts and methodology to the MSI domain, setting the stage to discuss approaches that stem from the interplay between these different ML paradigms and the acquisition, analysis, and interpretation of MSI data in the later sections. Readers are referred to prior literature^47,48^ for more in-depth and comprehensive accounts of various ML approaches, models, and algorithms.

### 3.1 Supervised Learning

The goal of a supervised learning problem is to learn a function (aka model) *f*_θ_(·) that can map the input data to a desired target value.^47^ A large set of paired data is typically used to train this model, denoted as *D* = {***x***_**i**_, ***y***_i_}_*i* = 1_^*N*^, where each ***x***_i_ is input data and ***y***_i_ is the corresponding “label” or “annotation” (note: we will interchange these terms in the sections below) for each ***x***_i_ (Figure 3). In the era of deep learning, the model *f*_θ_(·) is typically a multilayer neural network, parametrized by a set of weights and biases (denoted by θ), which are trained/estimated to establish the mapping between the input ***x***_i_ and the desired label ***y***_i_, such that ***y***_i_ ≈ *f*_θ_(***x***_i_). In the context of MSI, each ***x***_i_ may be the mass spectrum at each pixel on the tissue or an ion image extracted from the original multidimensional data **X̃**, and ***y***_i_ may be the corresponding tissue type classification (in the form of an index variable) or the whole-section segmentation mask (2D images). Alternatively, ***x***_i_ can be a truncated transient in FT-MS (discussed further in Section 4.2). In this case, the learning objective is to recover the full length transient, ***y***_i_ at a higher mass resolution (continuously valued 1D vector).

**Figure 3:**
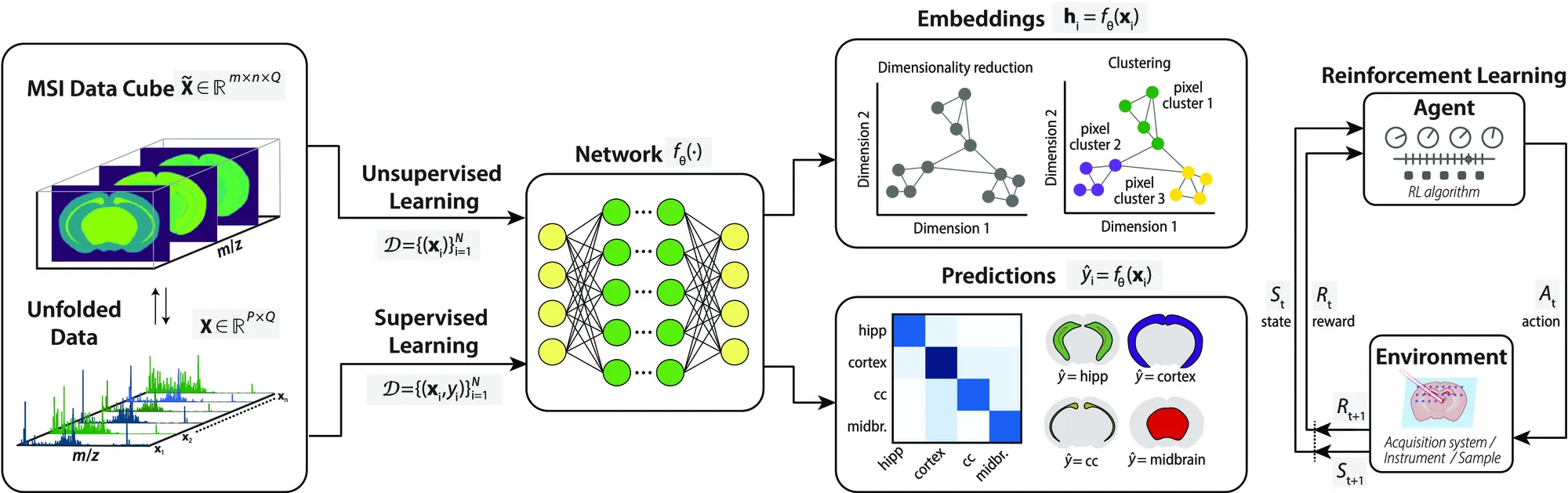
Illustration of different learning paradigms applicable to MSI. Multidimensional data **X̃** may be input as stacked ion images or unfolded matrices, per MSI sample. With unsupervised (or self-supervised) learning, embeddings/features can be learned from the data without “labels” for downstream tasks such as clustering. With supervised learning, data are paired with labels (e.g., higher-resolution counterparts, regions, or cell types). The models can be trained for classification (e.g., segmentation) or regression (e.g., image reconstruction) tasks. With reinforcement learning (RL), an agent learns to make sequential decisions based upon its environment. In the context of MSI, the environment can include the MSI instrument, sample, and acquisition software.

Supervised learning problems can be broadly categorized as classification and regression. In classification, the labels (***y***_i_) are discrete, e.g., ***y***_i_ = {1,2, ···, *C*}, and *C* is the number of classes. For instance, predicting whether an MSI pixel is tumor or nontumor is a binary classification problem,^49^ and in the example above, assigning each pixel to a specific tissue or cell type represents a multiclass classification task. In regression, the labels are continuous, e.g., $y_{i}\in R^{k}$ . In the example described earlier, predicting a full transient from a truncated one is a regression task where the target ***y***_i_ is a vector with continuous-valued elements. Another example is predicting ion intensities at unsampled pixel locations (either in a single ion image or jointly across multiple ion images). This can be viewed as a regression problem where ***y***_i_ would be formulated as either missing values or desired fully sampled images. In these supervised learning examples, the input data needs to be matched with a corresponding “ground truth” ***y***_i_ to train the model.

In many biological applications, obtaining a large quantity of paired data sets is challenging or even infeasible. For instance, consider the task of training a network to reconstruct high-spatial-resolution imaging data from low-resolution images. This would require thousands or more high-resolution data sets, each of which may take many hours or even days to collect (Section 2). Similarly, acquiring manual annotations of many tissue sections for a supervised classification or segmentation problem is both resource-intensive and time-consuming.

Strategies have been developed to address the limited labeled data challenge for supervised learning. One widely used approach is *data augmentation*, in which existing labeled data are augmented to produce additional training examples, e.g., via randomized rotation, flipping, cropping or intensity perturbations of the ion images, thereby increasing the effective size of the training data set without additional manual annotations.^50^ However, these augmentations do not truly enrich the biological heterogeneities across real samples. Another strategy is *transfer learning*, which assumes that there is a significant level of knowledge transferrable across modalities or tasks.^51^ In transfer learning, a baseline model is first trained on a large source data set (e.g., optical microscopy). The learned features and model parameters are then reused (transferred) and fine-tuned on a smaller target data set from a different modality (e.g., MSI).^52^ The source and target tasks may be the same or related (e.g., super resolution), similarly the baseline and target models may share their architecture either partially or fully. Empirically, low-level image features such as the edges and textures captured by the early layers of a deep network tend to be shared across modalities, while the high-level semantic features typically captured by the later layers are more modality-specific or task-specific.^53,54^ Therefore, during fine-tuning, it is often effective to selectively update certain layers while keeping others unchanged, which helps balance performance, generalizability, and computational efficiency.

### 3.2 Unsupervised Learning

In contrast to supervised learning, the unsupervised learning paradigm works without labeled data, i.e., learning a function *f*_θ_(·) using *D* but without paired ***y***_i_ for each ***x***_i_. A common example is dimensionality reduction: estimating lower-dimensional features (also known as the latent vector or variable), i.e., ***h*** = *f*_θ_(***x***), such that ***h*** preserves as much information as possible in ***x*** with a significantly lower dimension (Figure 3). One application of the extracted ***h*** is clustering, where similar data points (as defined by different similarity metrics) are grouped based on their inherent characteristics.^55^ This can then be used for a variety of purposes, e.g., exploratory analysis of high-dimensional mass spectra to mine similarity patterns, identifying unique tissue/cell types based on biochemical profiles, and segmenting MSI pixels into different biochemically defined regions.

While traditionally one may consider the goal of unsupervised learning is to uncover hidden patterns within the data via dimensionality reduction, its objective can be creatively designed. Particularly, **self-supervised learning** extends this paradigm by generating its own supervisory signals directly from the data through “pretext tasks” (tasks used for pretraining), such as interpolating missing image pixels, learning representations (or features of different scales) useful for various downstream tasks from vast amount of unlabeled data in this process.

Representation learning leveraging the power of deep neural networks has been expanding significantly in recent years. In self-supervised representation learning, a model is pretrained using a supervisory signal that is derived from the data itself through pretext tasks or data augmentation. One popular pretext task is masked modeling.^56,57^ Specifically, parts of the images will be masked, e.g., masking a number of pixels or even entire small patches (e.g., 8 × 8 or 16 × 16) in ion images, and a model is trained to predict the missing content. Interestingly, via this “interpolation” pretraining, the model learns to extract multiscale features (especially the encoder part) that can be later finetuned to a specific downstream task (often supervised). This has become a common approach for pretraining many large vision and language models.

Another self-supervised representation learning approach is **contrastive learning,** which teaches models to extract similar features from augmented versions of the same measurement/image while maximizing feature distance between different measurements/images.^58,59^ This enables the model to learn discriminative and generalizable representations of data without requiring manual annotations. Contrastive learning has been applied to MSI, either to ion images^60^ for discovering colocalized molecules, or to pixel-wise mass spectra^61^ for dimensionality reduction with maximum preservation of discriminative features and improved tissue segmentation.

Generative models, e.g., those based on generative adversarial networks (GANs) or diffusion models, can learn the underlying distributions of high-dimensional data in a self-supervised manner.^62^ Once trained, they can be used to synthesize realistic-looking samples, e.g., mass spectra, ion images, or transients, augmenting existing data or discovering hidden structures. Although synthetic data generation has not been well explored in MSI, it has shown strong potential in other modalities such as MRI^63^ and histological images.^64,65^ Considering the extensive resource and labor needed to annotate high-dimensional, high-resolution MSI data, generative model-based data synthesis may provide practical alternative to expand the training data for training more powerful deep networks, especially considering the rapidly improving performance of generative AI. Another promising direction is conditional generative models for image-to-image translation, where models are trained to synthesize new modalities from acquired ones. These generative AI-based image-to-image translation methods have received significant interest in various imaging domains, e.g., H&E to multiplexed immunohistochemistry (IHC),^66^ low-field to high-field MRI,^67,68^ and have potential in MSI, such as for generating MS images from histology or vice versa. In these strategies, potential bias introduced by complementary modalities and impact on downstream analysis need to be carefully assessed.

### 3.3 Semi-Supervised and Weakly Supervised Learning

Modern machine learning techniques often do not fall strictly into the discrete categories of supervised or unsupervised learning. Instead, they reside on a continuum with fully supervised and unsupervised learning at the ends of the spectrum. Many methods are designed to reduce the dependence on large quantities of manually annotated/labeled data. For example, **semi-supervised learning** combines supervised and unsupervised learning and learns from two sources of information: a small set of labeled data and a much larger pool of unlabeled data, where the labeled data guides the learning from the unlabeled data set. A semi-supervised method may use an unsupervised learning method to discover patterns or structures in the data, such as clusters, and then use a labeled data set to assign labels to the clusters.^69^ In MSI, mass spectra can be similarly clustered using unsupervised algorithms, and a limited number of annotated (tumor vs nontumor) spectra can then be used to assign labels to entire clusters.

Alternatively, a semi-supervised learning approach may first use labeled data to train an initial model and applied to an unlabeled data set to generate pseudo labels. The data with predicted labels that the model is sufficiently confident about are added to the pool of labeled data, and the model is retrained on the expanded data set. This can be repeated until the model’s performance converges. One example of this approach is an automated species identification method trained on a large MALDI data set with minimal labels.^70^ The importance of similarity metrics in semi-supervised learning, e.g., for identifying colocalized molecules, has been studied.^71^ It was demonstrated that with a proper choice of image similarity score, a semi-supervised DL model with a moderate quantity of expert-annotated ion image pairs can produce robust prediction in large unlabeled data.

Weakly supervised learning lies somewhere on the spectrum between semi-supervised and supervised learning, and applicable when the labels are imprecise or coarse in scale. For example, a whole tissue section may be annotated as “tumor,” even though an ion image from that tissue section contains both healthy and tumorous regions. Because the annotation does not precisely correspond to each pixel or region, the supervision signal is considered weak. A weakly supervised learning approach such as multiple instance learning can be used to classify the sublevel tissue location with tissue-level annotations which are weak labels.^72^

### 3.4 Reinforcement Learning

Reinforcement learning (RL) is another major learning paradigm, in which an “**agent”** learns to make sequential decisions to achieve a certain objective through trial-and-error interaction with an **environment (** Figure 3 **)**.^73,74^ The agent observes the current state *S*_t_ of the environment and selects an action *A*_t_ to perform. The environment responds to the action by transitioning to a new state *S*_*t*+1_ and providing a reward *R*_t+1_ that reflects the desirability of the outcome caused by the action. Through repeated interactions during training, the agent learns a policy that produces actions to maximize the long-term cumulative reward. This learning paradigm, if used properly, can aid in optimizing acquisition strategies and experiment design. Related work can already be seen in other imaging modalities that used RL to control an imaging system to adaptively balance measurement quality and acquisition time, e.g., X-ray imaging.^75^ Most MSI study designs currently involve significant manual choices by an analyst during the method development, optimization, data collection, and analysis stages. RL offers a potential systematic approach for searching the design space to minimize manual trial-and-error and subjective intervention, potentially leading to more efficient, robust and reproducible MSI workflows. Therefore, while RL has not been systematically investigated in MSI, we believe that developments in this direction carry huge rewards. We will discuss potential lines of pursuit in Section 7.

## 4 Data Acquisition/Reconstruction

Several data-driven learning-based computational approaches have been explored to enhance MSI data acquisition and reconstruction, improving imaging speed, spatial resolution, and mass resolution. In many examples, the data acquisition process is modified in synergy with the advanced computational methods. One important direction is accelerating data acquisition to enhance the throughput of MSI, especially for high-resolution instruments. We will broadly define the methods for accelerating MSI acquisition as “spatial acceleration” or “temporal acceleration”. In spatial acceleration, imaging speed is accelerated by reducing the number of pixels sampled while still covering the same tissue areas. For instance, this can be accomplished by sparsely sampling pixels within an imaging ROI and then reconstructing the remaining pixels with computational methods (Figure 4, **left panel**). In contrast, temporal acceleration is achieved by reducing the time of the MS detection window at some or all of the sampled pixels. As an example, this could be reducing the transient duration in FT-MS (Figure 4, **right panel**) and recovering high-mass-resolution data from the reduced transients via computational methods.

**Figure 4:**
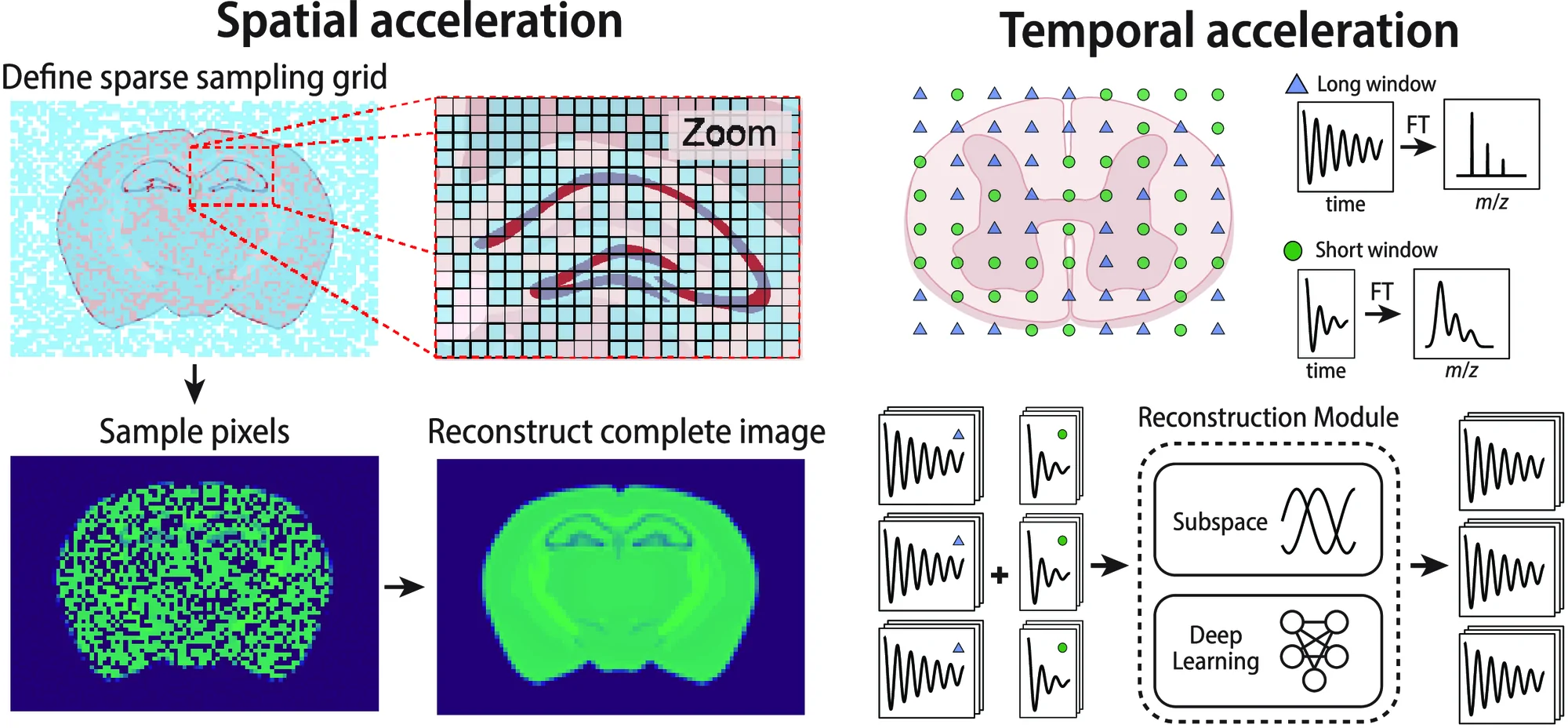
Approaches for accelerating the acquisition process in MSI. (Left) Spatial acceleration: MSI acquisition can be accelerated by sampling only a subset of pixels, here illustrated on a mouse coronal brain section. The missing pixels are then computationally reconstructed to recover the complete image. (Right) Temporal acceleration: a tissue section is randomly sampled with select long, high-resolution MS detection windows, while the majority of pixels are obtained with significantly shorter detection windows, yielding low resolution spectra, but decreasing imaging time. Using a subspace approach or deep learning, the short and low-resolution spectra are reconstructed to high-resolution spectra.

### 4.1 Sparse Sampling

The imaging time in MSI scales with the number of pixels/lines acquired (Figure 2), regardless of the mass analyzer used. Thus, one strategy to spatially accelerate MSI data acquisition is sparse sampling, where only a subset of pixels distributed across *the entire tissue section* is measured instead of uniformly sampling all the pixels. This subset of pixels may be selected from a predetermined random sampling pattern,^17,76^ a structured sampling pattern,^77^ or dynamically chosen from regions best predicted to improve image reconstruction.^16^ Computational reconstruction techniques are then applied to reconstruct the “sparsely sampled image”. We distinguish these approaches from those that reduce acquisition time by targeting predefined ROI(s) and still imaging with a standard raster-format (Section 6.2). One straightforward approach is to perform standard interpolation such as bicubic interpolation, which generally does not work as well as “model-based” image recovery algorithms combining a “data consistency” term and a regularization term imposing a certain image prior, e.g., a total variation (TV) regularization promoting piece-wise constant/smoothness of the unknown image. When the subset of sampled pixels are selected randomly, this falls under the umbrella of compressed sensing, a signal processing framework that enables recovery of high-dimensional signals from significantly fewer measurements by exploiting the inherent sparsity of a signal in a certain transform domain.^78^ Bartels et al. proposed a compressed sensing based formulation for MSI that simultaneously applies peak picking in the spectral domain and TV denoising in the spatial domain,^79^ showing proof-of-concept for recovering full ion images from fewer than half of the measurements. Xie et al. developed a practical method combining compressed sensing with subspace modeling (see Section 4.2) for the reconstruction of high-resolution FTICR MSI data.^17^ By acquiring only 30% of full pixel grids and transients of reduced duration, their approach achieved up to a 10-fold reduction in acquisition time. The reconstructed ion images largely preserved the spatial features compared to the fully sampled reference, with spatial correlations centered around 0.8 and peak-intensity correlation around 0.97, although some subtle structural details were not recovered, which remains a challenge for future computational method development. While these methods were not traditionally viewed as learning-based or AI-powered, they do fall in the broad category of *regression problems* in which each pixel or each raw transient point can be treated as a “label” for regressing a function to interpolate or extrapolate the missing points (pixels or transients).

With the rapid advances in deep learning, developments in image recovery and reconstruction algorithms have shifted toward deep network driven approaches, and MSI is no exception. Li et al. introduced DeepS, an algorithm combining a multiscale sparse sampling strategy with a multislice/3D image interpolation network (3D-SSNet).^80^ The network employs a generator and discriminator architecture trained with adversarial loss to reconstruct sparsely sampled ion images. The multiscale sampling strategy enables extraction of features at different scales for robustness to variations in tissue size and minimizing stage movement during acquisition. For training, tissue sections from one mouse brain evenly spaced throughout the data set were fully sampled and used for training 3D-SSNet in a supervised fashion, which was used to run inference on the remaining sparsely sampled sections. At 50%-pixel sampling, an SSIM (Structural Similarity Index Measure) of 0.79 was achieved (maximum is 1) compared to fully sampled images. At 5% sampling, the method was still able to reconstruct ion images; albeit with a SSIM of 0.64 and many fine details lost, indicating a limit on achievable acceleration rate. Xu et al. proposed a generative adversarial network (GAN)-based method that leverages both spatial and spectral information for missing pixel interpolation. More specifically, a deep autoencoder network was trained with both an MSE (w.r.t. to true pixel values) and an adversarial loss to interpolate within a local spatial neighborhood (“window-based”) simultaneously across multiple *m*/*z* channels. Structured sparse sampling pattern was used (unlike DeepS), i.e., using a larger raster width along each dimension with missing pixels in between, followed by local interpolation using the trained network. Improvements over traditional interpolation methods, including bilinear and bicubic interpolation in terms of SSIM and PSNR, were demonstrated.^77^

An approach was recently developed that integrates a raster scanning forward model and a plug-in, pretrained denoising network to reconstruct high-resolution ion images from sparsely sampled pixels.^76^ This method employs a plug-and-play framework that iteratively alternates between enforcing data consistency using the said forward model and applying regularization via the trained denoiser, producing reconstructed ion images with spatial correlations exceeding 0.9 for most ions at a 25% sampling rate, while achieving an average SSIM of 0.8 and an average PSNR of 28 dB at a 50% sampling rate. This method generalizes well across data sets acquired with different MSI instruments (FTICR and timsTOF), spatial resolutions ranging from 50 to 10 μm, and tissue types, including a brain section from an Alzheimer’s model mouse brain and rat kidney, without retraining the denoiser for each data set.

While the above methods work with predetermined sparse sampling coordinates, these locations can also be dynamically determined during the acquisition to optimize the trade-offs between time and accuracy. Hu and Laskin reported a dynamic sampling method to guide pixel selection during MSI on-the-fly.^16,81^ Their approach, termed Deep Learning Approach for Dynamic Sampling (DLADS), applied a prior dynamic sparse sampling algorithm developed for raster scans called SLADS (supervised learning approach for dynamic sampling)^82^ to MSI. Specifically, DLADS used network prediction to guide MS data acquisition to the pixel locations predicted to minimize reconstruction errors the most. DLADS was evaluated in both pointwise-acquisition mode such as MALDI-MSI and in line scanning mode such as nano-DESI (Figure 2). In a simulated pointwise sampling setting, DLADS adaptively selected a fraction of the pixel locations and yielded quality reconstruction via interpolation (i.e., 24.5 dB PSNR compared to the fully sampled image with only 10% pixels for a mouse kidney section). In a real line scanning experiment, DLADS was able to dynamically sample 40% of the total areas of interest (2.3-fold acceleration), one line at a time, and achieved reconstruction with strong consistency with the fully sampled image. We should note that SSIM and PSNR are popular general-purpose image quality metrics (e.g., an SSIM ≥ 0.8 generally indicates high visual coherence between two images). However, high SSIMs/PSNRs do not necessarily equate preservation of the chemical information desired. Expert evaluation in the context of specific applications is still needed. Task-driven quality assessment is an active research area in the broader field of computational imaging and is worth investigating in the context of MSI.

### 4.2 Accelerating Transient Acquisition

Beyond spatial acceleration by sparse pixel sampling, temporal acceleration can be achieved in FT-MS. FT-MS offers high chemical coverage, better mass accuracy and resolution of isobaric compounds. Despite its advantages, the long transients required for each pixel make it time-consuming. For example, imaging a single rat brain slice with one-second transients per pixel requires approximately 24 h of acquisition time. Consequently, FT-MS imaging is less suited for high-throughput applications, such as 3D imaging or clinical use.

To address this bottleneck, various strategies have been developed to increase imaging speed in FT-MS. Xie et al. introduced a subspace approach for FTICR MSI that exploits the low-rank structure in tissue MSI data,^83^ i.e., the ensemble of transients from an imaging experiment is treated as a low-rank matrix. This means that the desired long temporal transients, despite being high-dimensional, lie within a lower-dimensional subspace, which can be accurately estimated using a small random subset of full transients with the desired mass resolution (e.g., 10% or a couple thousand pixels) and captured by a small set of basis elements (Figure 4, right). With the subspace estimated, the remaining pixels were sampled with shorter transients (5% of the full length) and then extrapolated by least-squares basis fitting. This approach reduced the acquisition time by several fold while enhancing SNR and image contrast. As mentioned earlier (Section 4.1), this approach has also been combined with compressed sensing to leverage both spatial and temporal acceleration within the same imaging run.^17^ While not applied in the context of imaging, Kozhinov and Tsybin used a Lorentzian parametric model to fit FTICR data, achieving a mass resolution boost.^84^ Similar parametric approaches could be applied to accelerate FT-MS imaging, though the extensive computing involved for nonlinear parameter fitting needs to be considered.

To further improve the throughput of FT-MS at the single cell level and the 3D imaging regime, a deep-learning-based reconstruction approach termed MEISTER was introduced.^19^ MEISTER employs paired deep autoencoder architectures to learn low-dimensional features from raw high-resolution transient signals and to regress the low-dimensional embeddings from reduced transients. To realize 3D MSI, training sections 2 mm apart were imaged with long transients, generating high resolution data, while the remaining sections were acquired with only 5% of the full transients for enhanced throughput. When compared to the subspace approach, MEISTER reconstructed data with improved SNR, peak correlation, and spatial correlation (with full transients).

Both spatial and temporal acceleration strategies have great potential to improve MSI throughput and we envisage their increased adoption in the future as more high-resolution data sets are desired. While temporal acceleration has mainly been realized for FTICR, there are comparatively fewer examples for Orbitrap instruments, despite more widespread use. Although theoretical principles suggest that similar approaches could be applied to Orbitraps, software developments are necessary to enable the selection of pixels with short transients and to effectively process the raw data. The idea of temporal acceleration can also be applied to other modalities with time-intensive acquisition such as imaging experiments with nested ion-mobility. It is also worth noting that the computational acceleration techniques discussed here are complementary to advances in instrumentation and hardware. Looking forward, we see significant opportunities for combining spatial and temporal acceleration, facilitating fast, high-resolution MSI for 3D volumes, single-cell, and subcellular imaging, and clinical applications.

### 4.3 Image Super-Resolution

While reducing the pixel and/or probe size combined with sparse sampling can accelerate MSI, as the probe size decreases for higher-resolution scans, the amount of sampled material decreases at least quadratically, greatly reducing the signal strength. This signal reduction may be even greater as one also expects a decreased sampling depth as the probe area is reduced. On the instrumental side, several exciting developments have enabled MSI at impressive spatial resolutions while mitigating sample loss. One route of inquiry is the use of postionization lasers to improve analyte detectability when using high-spatial-resolution transmission mode geometries^7^ or microscope mode^42^ imaging.

In concert with instrumental developments, computational approaches to super-resolution are being developed. We consider computational super-resolution as a powerful approach to accelerate MSI acquisition while minimizing signal penalty, rather than replacements for instrumental advances that enable measurements near the physical resolution limits of MSI. Instead of distributing the pixels sparsely across the tissue sections, a larger probe size is used during scanning than the final desired resolution (with stronger signal per pixel), reducing the number of pixels needed and effectively accelerating the acquisition. Image super-resolution techniques can then be applied to reconstruct ion images with finer grids from the lower-resolution counterpart, providing finer spatial detail without increased acquisition time, signal penalty, or other experimental challenges.

We broadly define existing approaches for MSI super-resolution in three categories (Figure 5a-c). These approaches are not mutually exclusive, and many emerging methods integrate elements from more than one. The classic model-based methods formulate the sampling problem by treating each pixel as a spatially blurred measurement and solve an inverse (deconvolution) problem.^85,86^ Although extensively used in imaging modalities such as optical microscopy, these methods have not seen much adoption in MSI as the beam profile or tissue ablation pattern (aka blurring kernel) can be difficult to measure and model mathematically for many instruments. Cross-modal approaches enhance spatial resolution by leveraging higher-spatial resolution data from another modality, such as optical microscopy. These images provide structural and morphological detail to sharpen MSI data, with approaches including image-patch-based synthesis, multivariate regression, and deep learning.^18,87,88^ In learning-based super-resolution, deep neural networks are trained to infer high-resolution MSI from a lower-resolution input. As one example, large quantities of paired high-resolution and low-resolution optical microscopy images can be leveraged for supervised training of the super-resolution network followed by transfer learning (Figure 5d).^52^ We will discuss some representative works from these approaches below.

**Figure 5:**
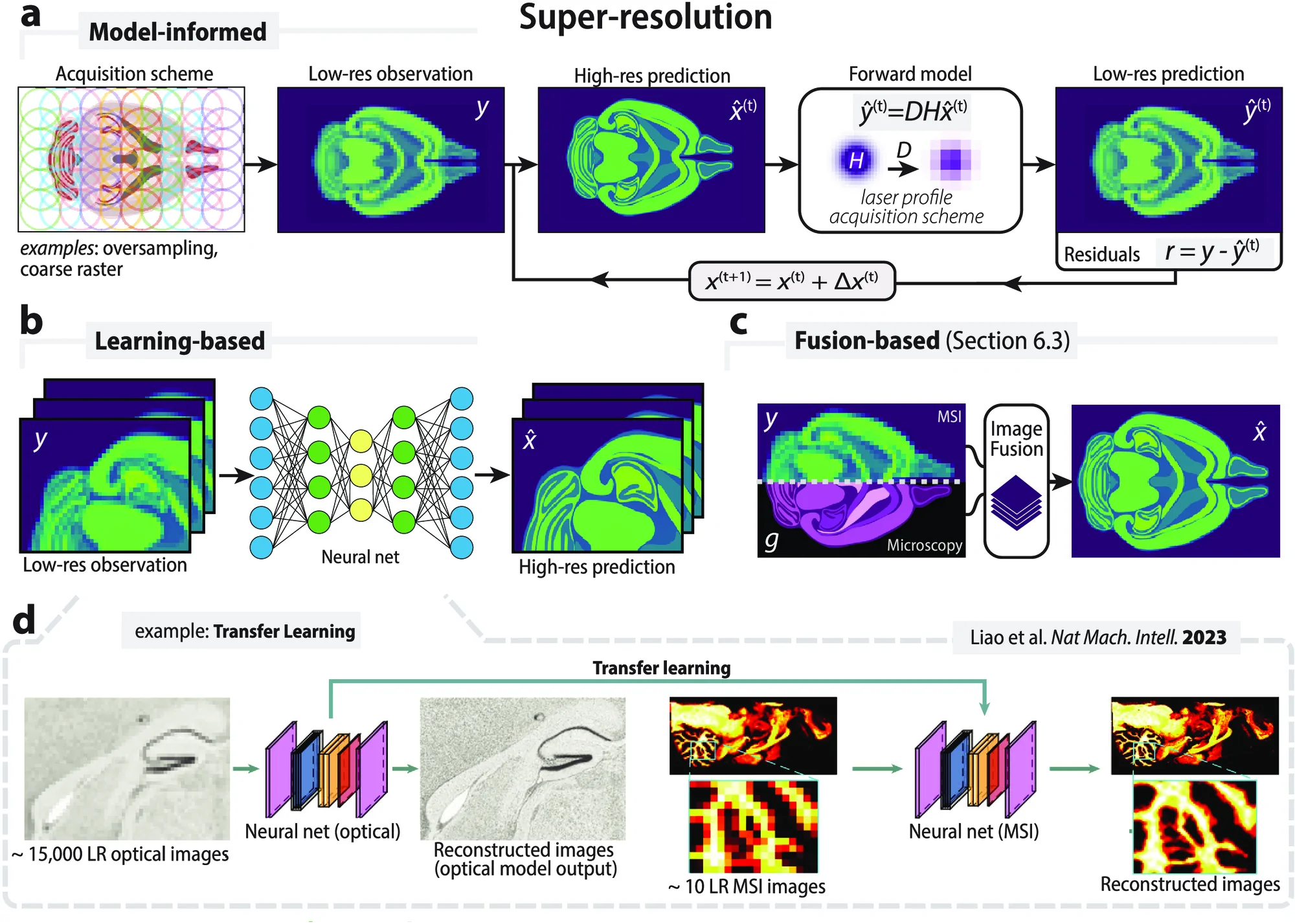
Image super-resolution approaches applied to MSI. (a) Model-based methods model the sampling process (first image) and apply deconvolution to recover high-spatial-resolution data that is consistent with the low-resolution counterpart. (b) Learning-based methods train a model (deep networks) to generate high-resolution data from low-resolution inputs. (c) Cross-modal approaches leverage images with higher spatial resolutions from another modality to sharpen MSI data. (d) An example of learning-based approach: the idea of *transfer learning* was applied. An image super-resolution network was first trained with abundant optical images. The learned features and model parameters were then transferred to and fine-tuned using MSI data. Adapted with permission from Liao et al. *Nat. Mach. Intell.* (ref.,^52^ Copyright 2023, Springer Nature).

As an example of model-based methods, Van Malderen et al. reported a super-resolution method using oversampling (Figure 5a) combined with deconvolution.^86^ The authors formulated and solved a deconvolution problem using a blurring kernel derived from post-MSI measurements of the ablation craters to recover pixels less than the probe size. In contrast to oversampling with complete ablation,^89^ this approach can, in principle, generate images with resolution higher than that achievable by the minimum stage movement and probe size. More recently, a model-based method using oversampling was applied to resolve subcellular structures with imaging mass cytometry.^90^ The authors modeled a point spread function (PSF) based on overlapping circle geometry, working in a laser energy regime that preserved signal over multiple passes. The oversampled images were then reconstructed to super resolved images using Richardson-Lucy deconvolution. In our recent work, we demonstrated that deconvolution can effectively enhance the spatial resolution of MALDI-MSI, with varying degrees of oversampling.^76^ Each low-resolution pixel, acquired with a relatively large laser spot (or probe size), can be modeled as a local convolution of neighboring high-resolution pixels. A plug-and-play framework was employed to combine the model-informed reconstruction and a deep network based denoising prior, using a local averaging kernel based on post-MSI ablation craters. This approach outperformed standard interpolation techniques such as bilinear interpolation with more accurately recovered fine structural details.

For cross-modal super-resolution, several methods have been proposed to leverage a “higher-resolution” modality to help boost the resolution of MSI data.^18,24,87^ Van de Plas et al. proposed to use H&E-stained microscopy images to enhance the spatial details in MSI data via a multivariate regression approach,^18^ and reported a resolution enhancement of 10-fold or more. Ščupáková et al. developed a patch-based super-resolution algorithm that uses high-resolution features extracted from patches of optical microscopy images to guide a super-resolution reconstruction of ion images from MSI.^87^ Several other more extended super-resolving MSI methods have been put forward lately, which fall into the general category of multimodal image fusion. We will discuss these methods in greater detail and depth in Section 6.3.

Learning-based methods have been widely applied to super-resolve images in various modalities. A deep network-based model trained on a large data set can be used to enhance the spatial resolution of ion images. However, unlike optical microscopy, supervised training in the MSI domain is challenging due to the limited number of data sets/sections with matched desired higher resolutions. To address this challenge, Liao et al. proposed a transfer learning–based approach called MOSR (MSI from Optical Super-Resolution) (Figure 5d).^52^ This approach leverages the abundance of optical microscopy images (∼15,000) for initial supervised training. The model is then fine-tuned using a limited set of MSI data, enabling it to learn MSI-specific patterns. MOSR achieves up to 4 times spatial resolution enhancement and outperforms direct supervised learning approaches in reconstruction quality, in terms of image correlation, PSNR, and SSIM. In addition to improved performance, MOSR enables faster training and exhibits strong generalizability across different MSI modalities, tissue types, and organisms.

These computational strategies continue to evolve rapidly, offering approaches for spatial resolution beyond the physical limits of the MS probe size. In other cases, they can be used to accelerate data acquisition and improve signal quality for imaging at moderate resolution. We anticipate that super-resolution techniques will become increasingly integrated into MSI workflows, enabling more detailed spatial-molecular characterization across a broad range of applications.

## 5 Data Analysis and Interpretation

With the multidimensional nature of MSI data and rich biomolecular information therein, a diverse set of processing and analysis steps may be applied to derive chemical and biological information. Data-driven ML- and AI-powered methods play critical roles at these steps. In addition, many preprocessing steps need to be performed to prepare the raw MSI data for subsequent advanced computational analysis. This section reviews key methodologies and recent advances on these steps, including preprocessing, dimensionality reduction, and segmentation and higher-level analysis. In each area, we highlight the application of computing and AI to streamline data analysis, identify subtle patterns missed by manual or conventional tools, and improve the interpretation of high-dimensional MSI data sets.

### 5.1 Conventional Processing Strategies

#### 5.1.1 Standard Preprocessing Steps

Raw MSI data sets are exceptionally large volume, high dimensional, and yet intrinsically sparse in the *m*/z domain, making preprocessing and denoising indispensable before meaningful analysis and biological interpretation can occur. Significant data engineering efforts have been made to extract raw imaging data and convert them from vendor-specific file formats into language-specific or open-source formats. A landmark development is the open-source .imzML format tailor-made for MSI^91^ and most vendors now allow data to be exported from commercial software into .imzML, paving the way for developing advanced ML/AI-based tools. (Note: Vendor software may already perform some preprocessing behind the scenes which the users might not be aware of and can impact methodology development. Fortunately, API is available for most vendors that allow wrappers to be developed for parsing and exporting raw data. Examples include pyTDF-SDK for Bruker timsTOF,^92^ Spike for Bruker FT-MS and Thermo Orbitrap,^93^ and rawrr for Thermo Orbitrap.^94^) During acquisition, some “on-the-fly” preprocessing often occurs, such as summing successive spectra (TOF “histogramming”),^95^ peak centroiding (if in centroid mode), lock-mass calibration, and so forth.

Once raw (or minimally preprocessed) data are extracted, researchers and practitioners often proceed with some or all of the “standard” preprocessing steps, which are illustrated in Figure 6. In this section, we will provide a brief summary of these steps, their inherent challenges, and review emerging ML/AI tools that can improve and even “replace” these often tedious, operator-dependent steps. Readers who are interested in a more detailed review of preprocessing methods and tools for MSI can refer to several excellent reviews on the subject.^96,97^

**Figure 6:**
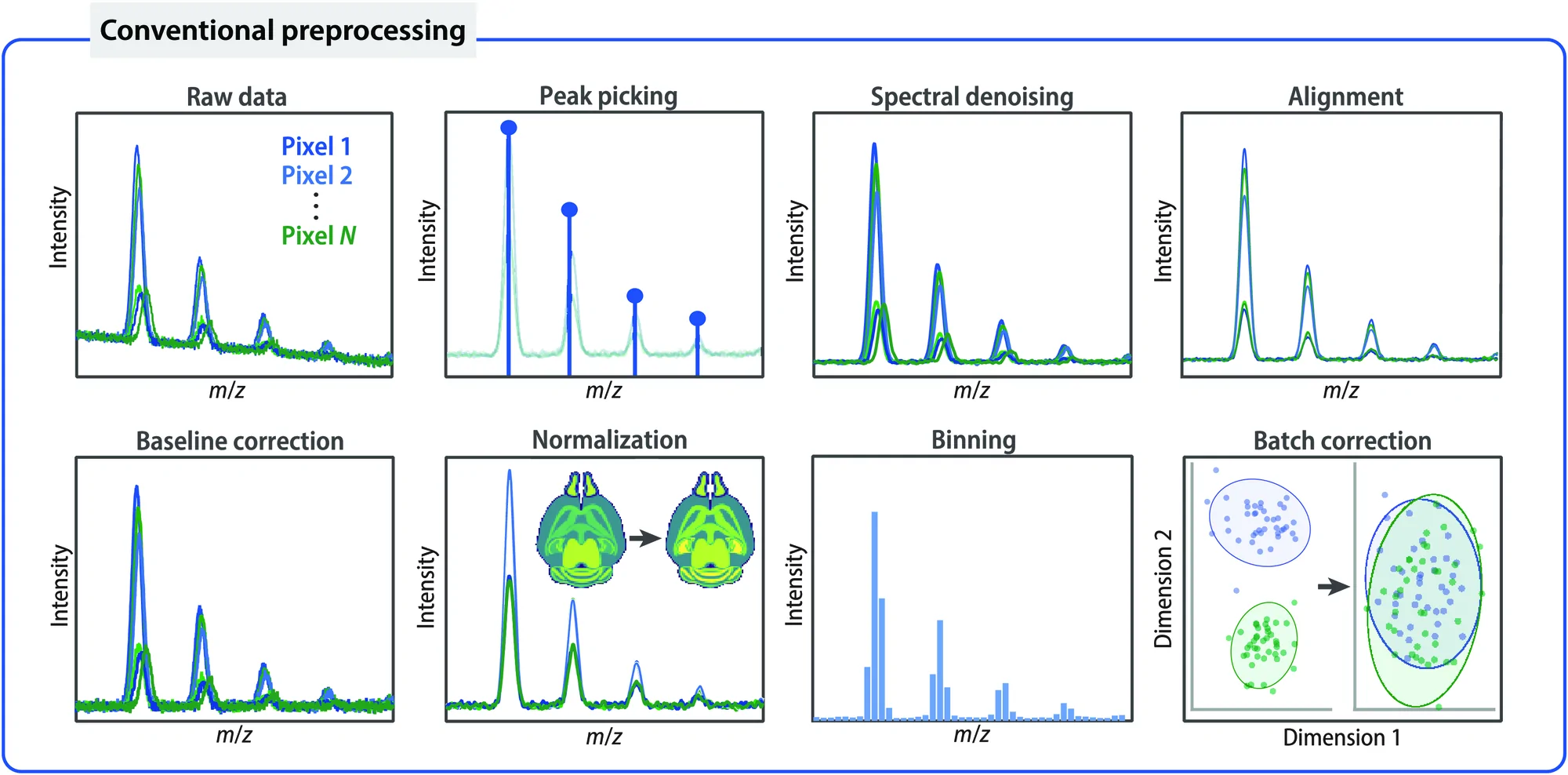
Preprocessing steps applied to MSI data. The first panel illustrates the raw data for a set of *m*/*z* features, with each trace representing a unique pixel. Depending on the instrument and application, various preprocessing steps including baseline correction, spectral alignment, and denoising (either in the spectral or spatial domain) may be performed.

*Peak picking* is typically a required step. MSI data at each pixel is inherently sparse. Although the raw spectral signal can have tens of thousands to even millions of points, signals are concentrated in narrow *m*/*z* windows, with chemical, detector, and shot noise occupying the regions in-between. Therefore, peak picking is often used to identify *m*/*z* peaks, eliminating noisy or chemically uninformative regions within the spectrum and effectively reducing dimensionality to a few hundred or thousand *m*/*z* channels. Traditional approaches use local maxima detection combined with intensity thresholds to distinguish true peaks from noise. Recently, more powerful approaches have leveraged the spatial-spectral information inherently provided by MSI to provide more robust peak identification. For instance, Lieb et al. developed a peak detection algorithm that uses sparse frame multipliers leveraging both spatial and spectral information for peak selection.^98^ Specifically, this approach enforced consistent presence of a given peak (ions) in a small spatial neighborhood to improve robustness.

*Binning* in the spectral domain is commonly applied after, or in place of, peak picking. Binning serves three purposes. First, during the course of MSI detection, the exact detected *m*/*z* of a particular peak may change between pixels and runs due to the statistical nature of ion detection or instrumental drift.^99^ Distributing all data to a common set of bins accounts for this uncertainty and generates consistent *m*/*z* arrays of constant size across all pixels. Second, most mass spectrometers obtain data with a sampling rate yielding several points across an *m*/*z* peak. Summation of these points into a single or fewer bins improves the SNR of the final ion image. Third, binning reduces data size, simplifying downstream processing. While binning is an essentially ubiquitous step in MSI preprocessing, a few interesting alternatives have been proposed in the broader mass spectrometry space. De Jonge et al. trained ML models using set-based and graph-based representation of spectra rather than fixed-length, binned data.^100^ While this was demonstrated on tandem MS data, it has not been applied to MSI, for which there might be significant memory and computation issues. Pérez-Cova et al. developed a combined peak picking and binning routine, termed MSroi, which identifies informative *m*/*z* regions based on criteria of signal intensity, a user-specified mass tolerance, and the number of occurrences for that mass across pixels.^101^ This preprocessing approach addresses the unequal length of *m*/*z* and intensity pairs between pixels, through user-supervised preprocessing in a straightforward GUI, facilitating advanced downstream data analysis.

After peak picking and binning, *normalization* is commonly performed and is especially important for sample-to-sample and group-to-group comparisons. We distinguish between intrasample, or pixel-by-pixel normalization, and intersample normalization. Approaches for pixel-by-pixel normalization have been discussed and reviewed in detail.^26,97^ Deininger et al. provide an excellent description of the benefits and drawbacks of common normalization schemes applied to MSI.^102^ While commonly applied, TIC-normalization can introduce spatial artifacts into MS images. For instance, if the TIC image shows strong white-matter-associated structure because many abundant ions are enriched in white matter, applying TIC normalization can “burn” this pattern into low intensity *m*/*z* features.

To address the shortcomings of TIC normalization and similar strategies, internal standards can be used, especially for targeted normalization of specific analytes or compound classes. For example, Lanekoff et al. normalized lipid signals in nano-DESI MSI to that of an internal standard added into the solvent, recovering accurate spatial distributions.^103^ Expanding on this concept, Vandenbosch et al. demonstrated an omics-scale quantitative approach relying on a preacquisition physical preparation: spraying a multiclass IS mixture homogeneously over the tissue.^104^

Another normalization strategy introduced is the use of so-called tissue extinction coefficients (TEC). Briefly, a standard is evenly sprayed onto a reference tissue section and the slide. The TEC is then calculated as the ratio of intensity for an analyte between a specific tissue region relative to the intensity on the glass slide.^105,106^ It should be noted that while both internal standard and TEC normalization help address region-specific ionization suppression, both approaches assume uniform analyte extraction across the tissue and do not necessarily address differences in how analytes extract between different tissue environments such as a cell membrane, cytoplasm, or organelle. Therefore, region-dependent matrix effects can still persist despite normalization.

*Spectral denoising* is generally helpful, especially considering the trend toward decreasing pixel size in modern MSI acquisitions to improve spatial resolution but associated with decreasing signal-to-noise ratios (SNR). Denoising can be as simple as a linear filtering step (such as exponential filtering of the transient data from an FTICR instrument) before peak picking, or more sophisticated AI-powered denoising using deep neural networks (see Section 5.2.1). In some cases, *spectral alignment* may be performed. In most cases, shifting of *m*/*z* peaks is insignificant, and minor amounts of shifting are addressed during binning and/or with lock-mass calibration. However, for studies using older generation instruments, or integrating data collected at different times, an explicit alignment step might be helpful. Eriksson et al. developed an alignment algorithm for peak-picked/centroided MSI data based on correlation optimized warping (COW),^107^ which was originally developed to align chromatographic data by piecewise stretching and warping.^108^ They applied their approach to MALDI and nano-DESI ionization sources and TOF and FT-MS mass analyzers.

Finally, as the application of MSI grows to larger sample cohorts and 3D imaging, intersample normalization and addressing *batch effects* are becoming increasingly important. Balluff et al. provide a detailed evaluation of strategies to recognize and account for batch effects in MSI data sets.^109^ Recently, Metodiev et al. comprehensively assessed several batch correction algorithms for their usefulness in correcting samples analyzed on 10 slides over 10 days of analysis.^110^ Huang et al. used tissue-mimicking standards (propranolol embedded in gelatin) to simulate ion suppression behavior in real tissues. By preparing samples on three different days, the authors generated three “batches” of data, and compared normalization and batch correction strategies, finding the best improvement achieved using internal standards for normalization and NormAE (a deep autoencoder approach)^111^ for batch correction.^112^ In one strategy applied to single cell MSI, Bessler and co-workers added a dye to healthy control samples to all slides in order to differentiate them from other sample groups. Then, given that healthy controls should not vary slide-to-slide, the authors could use ComBat effectively and without removing real biological variability.^1000^ These studies highlight that the use of control standards or samples, along with correction methods such as ComBat and NormAE, is emerging as a promising solution to mitigate batch effects in MSI data sets.

With the rising popularity and success of ML/DL-based methods in various imaging modalities, enhancing the preprocessing and analysis steps for MSI via data-driven methods have attracted intense research interest.^107,113^ We expect to see more exciting progress on this front, potentially combining several preprocessing steps into one strong model with improved automation and robustness.

#### 5.1.2 Quality Control

Data *quality control* (QC) is a key issue in MSI worth discussing. Data outliers and measurement drifts can substantially confound biochemical information of interest and negatively impact downstream analysis. QC and data correction methods tailored to MSI are being developed. For instance, Sohn et al. reported a workflow wherein they first sprayed an analyte mixture onto a slide, imaged the standards, and used principal component analysis (PCA) with statistical thresholding to determine if the MSI system is still suitable.^114^ Likewise, the tissue mimetic study by Huang et al. discussed above shows how tissue-mimicking standards can serve as QC samples.^112^ ML/AI approaches can be effectively used for QC, e.g., measurement drifts and batch effects may be detected by inspecting the spread of embeddings from dimensionality reduction (Section 5.1.3), which can be hard to accurately describe using conventional analytical approaches with “hand-crafted” features.

#### 5.1.3 Dimensionality Reduction

After peak picking and binning, MSI data still may contain millions of pixels and thousands of *m*/*z* features per spectrum. This makes interpretation challenging. Suppose one wants to determine chemical compounds that distinguish samples/experimental groups, identify sample outliers, or identify spatially correlated *m*/*z* features, one approach might be to compare the intensities of each *m*/*z* feature, one-by-one, between different samples. However, the sheer enormity of the data makes this strategy too labor-intensive and even prohibitive, especially with large sample sizes in biological/clinical studies or 3D imaging. Another problem is that when performing univariate analyses like this, any covariation between variables (e.g., *m*/*z* or pixels) is explicitly ignored.

***Dimensionality reduction*** addresses this issue by transforming high-dimensional data into a low-dimensional representation that retains meaningful relationships within the original data and is easier to interpret. The simplest form of dimensionality reduction is “manually” selecting a subset of targeted *m*/*z* features, which can introduce significant bias. In the past decades, several computationally driven approaches were developed, including prototypical linear and more advanced nonlinear methods. Below, we review major dimensionality reduction approaches and their application to MSI data. Then, we explore computational strategies to improve the scalability (speed and memory efficiency) of dimensionality reduction approaches.

Linear methods such as PCA have been described and reviewed in the context of MSI previously,^115^ and an intuitive tutorial on how PCA works is nicely illustrated by Bro and Smilde.^116^ In essence, most linear dimensionality reduction methods can be viewed in the form of matrix factorization, with the statistical and mathematical interpretations differing for each method (illustrated in Figure 7a). Consider an MSI data cube unfolded as a matrix $X\in R^{P\times C}$ of *P* pixels and *C m*/*z* channels (after peak picking). PCA finds a new set of orthogonal axes, termed the principal components (PCs), in an *L*-dimensional subspace embedded in the original high-dimensional space. The PCs are constructed such that the first PC captures the dimension with the most variance in the data, the second PC captures the second most, and so on. The useful outputs of PCA are two low-rank matrices, scores ( $U\in R^{P\times L}$ ) and loadings ( $V\in R^{L\times C}$ ). Each column of the scores can be refolded into an image that captures spatial patterns of variance, and each row of the loadings forms a pseudospectrum, where positive values are correlated and negative ones are anticorrelated. If most of the variance is captured by the first few PCs, the scores provide a useful pixel-wise low-dimensional embedding. Scatter plots of the scores on PC1 vs PC2 can help visualize underlying relationships in the data (Figure 7d). PCA is widely used in MSI because it is simple, generally interpretable, and deterministic (running PCA multiple times always yields the same result). Meanwhile, PCA assumes that the data are Gaussian distributed which may not hold for biological data. *Independent component analysis* (*ICA*) finds a set of components which are statistically independent, rather than following a specific distribution such as Gaussian. In contrast, non-negative matrix factorization (NMF) assumes that each spectrum is a combination of a small set of *non-negative* components with minimal statistical assumptions, respecting the physical nature of MSI data — ion intensities cannot be negative.^117^ Unlike PCA, there is no closed-form solution for ICA or NMF and the estimated components depend on choices in the number of components, initialization strategy, and regularization parameters. Interested readers may refer to Verbeeck et al.^13^ on various algorithms for matrix factorization.

**Figure 7:**
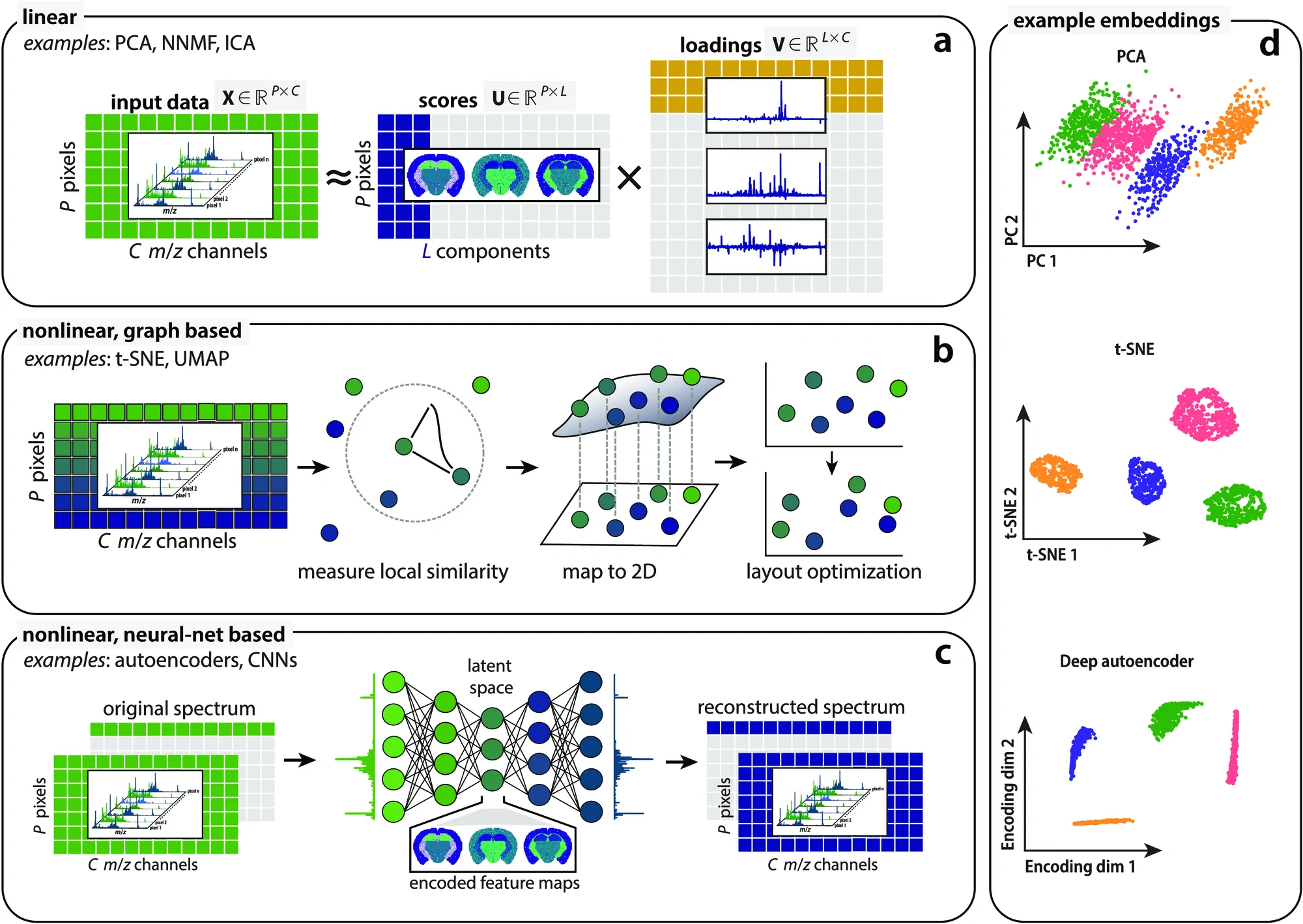
Dimensionality reduction approaches applied to MSI data. Broadly, approaches are categorized as (a) linear, (b) nonlinear graph-based, and (c) nonlinear neural network-based techniques. Trends in the data can be visualized by scattering the embeddings, shown in (d).

Linear models selected and adapted to the unique data characteristics in MSI have been proposed. Moens et al. reported a low-rank model with sparse and dense residuals.^118^ In standard PCA, data are factored into the low-dimensional subspace and Gaussian residuals (gray elements in Figure 7a), which is not good at capturing uncorrelated but sparse components. The low-rank and sparse model (also referred to as robust PCA)^119^ attempts to capture localized sparse signals (such as artifacts and rare biological features), and makes the final low-rank approximation less skewed by these deviations versus standard PCA. It was demonstrated that spatially sparse signals were effectively captured by the sparse component, giving users the flexibility to include these signals in the low-dimensional subspace if desired, or exclude them if they are artifacts.

While linear dimensionality reduction methods are computationally efficient and generally more interpretable, they often cannot identify meaningful patterns in high-dimensional biological measurements which are not linearly separable.^120^ Nonlinear dimensionality reduction methods can better capture such nonlinear structures (“manifolds”) and often separate meaningful biological variation from technical artifacts more effectively than linear methods. In the early 2000s, a growing body of work focused on preserving distances within a local neighborhood on the manifold where the data effectively lie. Van der Maaten and Hinton introduced t-SNE in 2008 as the culmination of a series of earlier methods,^121^ and was it rapidly adopted by the scientific community. T-SNE was first applied to MSI data in 2012 by Fonville et al., who used clusters in t-SNE space (from pixel-wise data) to generate RGB-color coded images that represented most of the variation in the data sets, thus providing anatomically rich representations of MSI data.^122^ They reported that this approach revealed finer tissue biochemical differences than PCA.

The UMAP (Uniform Manifold Approximation and Projection) technique rapidly received widespread adoption after its first publication, due to faster runtimes vs t-SNE and the *perception* that it better preserves global structure (though this is not necessarily always the case).^123^ UMAP was first applied to MSI in 2019 and has been used extensively since.^55^ Nonlinear, graph-based approaches, such as t-SNE and UMAP, first compute a matrix of pairwise similarities (a neighborhood graph) in the original high-dimensional space (Figure 7b). The data, assumed to lie on a low-dimensional manifold, are then mapped to a low-dimensional embedding, usually two or three dimensions for visualization. The layout of the embedding is iteratively optimized via (stochastic) gradient descent such that the embedding preserves the high-dimensional neighborhood relationships. For those interested in further details, see Kobak and Berens for an illustrative example of how to use t-SNE^124^ and Healy and McInnes for a tutorial of how UMAP works.^125^

Recently, multilayer neural networks are emerging as a major computational mechanism for nonlinear dimensionality reduction in many imaging modalities.^126^ Their use in MSI has also seen increased popularity in recent years.^120^ The archetypical example of these is the autoencoder, as shown in Figure 7c. For an autoencoder trained on spectra, data at each pixel is mapped into a low-dimensional latent vector. For any given network unit (aka neuron) in the hidden layer, collecting its activation across all pixels and refolding back to an image yields encoded feature maps analogous to a PCA score image. Note that the fully connected-like layer in Figure 7c is only a simplified illustration. The network layer can be convolutional (e.g., for ion images as input), or graph-based with each node capturing “higher-level” features such as spectra or image intensities. These different types of network layer designs for MSI data were nicely summarized in a recent review by Beck et al.^120^ Unlike graph-based methods, for which the local coordinates need to be recomputed when new data points are included, the networks, once trained, can run inference on new data with efficient computation.

The application of nonlinear, network-based dimensionality reduction to MSI was first reported by Thomas et al., who applied a fully connected variational autoencoder to mouse brain data.^127^ They presented evidence that an autoencoder could recover anatomical structure better than PCA, demonstrating the effectiveness of autoencoders for extracting salient low-dimensional features from MSI data. Later, Abdelmoula et al. expanded on this work, similarly using pixel-wise MSI data as input, further mapping latent features back to input layers to identify cluster driving *m*/*z* values in each class.^113^ This approach was applied across large-scale 2D and 3D MSI data from several different instrument platforms. Most recently, convolutional autoencoders (CAE) have been applied to TOF-SIMS data.^128^ CAE was designed to take images as input, and may be a more powerful feature extraction mechanism for 2D spectra and/or spatial-spectral input than conventional autoencoders that only take one-dimensional input.

We next review two key directions in the development of dimensionality reduction for MSI: (1) a large body of work focused on making dimensionality reduction faster and more scalable to increasingly larger data sets; (2) how dimensionality reduction is combined with high-level downstream analysis tools, such as segmentation and clustering, to extract meaningful biological information from MSI data sets.

#### 5.1.4 Scalability: Speed and Memory Efficiency

Modern MSI instruments and experiments often generate large data sets that pose significant challenges for practical implementation of dimensionality reduction, i.e., long computation times and high memory requirements. Generally, with the major exception of neural network-based methods, dimensionality reduction tools require the entire data set to be loaded into a computer’s RAM, which can exceed 100 GB. This has motivated the development of more efficient computational approaches for dimensionality reduction for MSI data. In one of the first approaches, Race et al. applied a reformulated PCA approach so that the covariance matrix is built sequentially from individual spectra, rather than loading the full data set into RAM.^129^ This enabled PCA of data sets exceeding 40 GB without discarding pixels or peaks, preserving full data fidelity, at the expense of longer runtimes. Their method was demonstrated on MALDI MSI data sets while in principle it is applicable to other modalities. Cumpson et al.^130^ extended scalability strategies to ToF-SIMS by applying the “randomized vector” method of matrix decomposition developed by Halko and co-workers.^131^ These methods use random subsets of columns or rows from a large matrix to identify a subspace that captures most of the important structure. This allowed full-resolution PCA without coarse binning or restriction to regions of interest, and in one case revealed NaCl microcrystals on sodium citrate crystals that would have been missed using manual analysis of individual spectra.

Krijnen et al.^132^ advanced this line of work by applying optimized incremental PCA to MSI. Incremental PCA (IPCA) loads data in batches, generates principal components, which are then updated as more batches of data are added. The authors evaluated this approach across implementations of IPCA in Python, and Rust, and compared to standard PCA as performed in commercial software like SCiLS lab. Interestingly, the authors observed that IPCA performed faster than PCA despite requiring more computations to complete. The authors observed that the runtime significantly increased after selecting a particular number of batches, suggesting that the batch size had an effect on the speed of the singular value decomposition itself. Further, they hypothesized that loading smaller batches of data into RAM, as done in IPCA, is more cache friendly and could contribute to improved runtimes.

Collectively, these studies illustrate a trajectory in linear dimensionality reduction development from runtime optimization^133^ to memory-efficient reformulation,^129^ random matrix sampling for large surface data sets,^130^ and fully incremental implementations for next-generation instruments.^132^ Together, they show how linear methods are being adapted as a practical and scalable tool for MSI analysis, with approaches designed to extend to the terabyte scale. These advances highlight how algorithmic innovations drawn from the broader computing and data science community are reshaping MSI workflows, enabling analyses that would have been computationally infeasible little more than a decade ago. While most efforts to improve scalability have focused on PCA, other methods have also been benchmarked with attention to runtime and memory efficiency. For example, Nijs et al.^134^ evaluated multiple NMF formulations from a computational standpoint, highlighting differences in convergence speed and efficiency across variants. They noted that “sparse” NMF models, while faster, significantly suffered in performance. Approaches to improve speed and scalability have also been developed for nonlinear dimensionality reduction methods. In 2014, van der Maaten reported tree-based algorithms for minimizing the KL-divergence loss in deriving t-SNE embeddings, particularly the use of Barnes-Hut approximation of the gradient (BH t-SNE).^135^ Their algorithm reduced the computation complexity from *O*(*P* ^2^) to *O*(*P* log *P*) in the standard t-SNE, where *P* is the total number of pixels. BH t-SNE is generally considered to be a faithful approximation and is the default version of t-SNE implemented in many packages, such as scikit-learn. Smets et al. achieved superior runtimes for UMAP applied to MSI data as compared to t-SNE and Barnes-Hut t-SNE, with *O*(*P* ^1.14^) complexity.^55^

While dimensionality reduction is a powerful, and often crucial step in the MSI data analysis workflow, it is seldom sufficient on its own. It may enable downstream data analysis that would not be possible to apply directly on the raw data, or it can significantly simplify and accelerate the data analysis process. Next, we review how dimensionality reduction tools, combined with high-level/downstream analysis and powered by advanced ML/AI tools can enhance segmentation, tissue classification/identification, and data quality, to facilitate the interpretation of MSI data.

### 5.2 ML/AI Augmented Data Analysis

#### 5.2.1 Segmentation

A key step for MSI following preprocessing and dimensionality reduction is *segmentation*. Spatial segmentation/parcellation is crucial for capitalizing on the rich spatially organized molecular information provided by MSI and extracting biological insight buried within such high-dimensional data.^3^ In contrast to conventional anatomy-based segmentation, we focus here on segmentation driven by tissue-specific biomolecular profiles that enable discovery of biologically or pathologically relevant regions of interest (ROIs). Data-driven segmentation is typically performed by *clustering* mass spectra from different pixels based on their similarity. In some cases, this is done in the space of low-dimensional embeddings (Section 5.1.3). We first discuss conventional, ML-based approaches and then explore a range of advanced deep learning methods.

A number of clustering algorithms, e.g., k-means, Gaussian mixture models (GMM), and hierarchical clustering, have been applied to pixel data in MSI post dimensionality reduction. Each reduction method may emphasize different aspects of the data structure and distribution; thus, identifying optimal pairing is challenging. A comparison study by Prasad et al. evaluated several state-of-the-art clustering methods, including k-means, spatially aware variants, and GMM, for MSI segmentation.^136^ These methods were evaluated in combination with different dimensionality reduction methods, including PCA, t-SNE, and minimum noise fraction (MNF) transform, to determine which combination is best suited for capturing biochemical patterns in MSI. It was shown that k-means clustering with correlation distance and GMM, most faithfully recovered ground truth cluster assignments from simulated data with a moderate number of *m*/*z* features. However, performance on real MSI data was assessed only qualitatively, and the authors noted that MNF does not scale well to high-dimensional spectra (>1000 *m*/*z* features)^136^

On the other hand, an ensemble strategy can mitigate the limitations of any single approach. Hu et al. proposed a method that combined multivariate clustering and univariate thresholding to improve the quality of tissue MSI segmentation.^137^ In the *multivariate module*, PCA was used to generate pixel-wise dimensionality reduced features for GMM-based clustering. In parallel, UMAP followed by HDBSCAN (Hierarchical density-based spatial clustering for application with noise) was also explored. The authors suggested the use of UMAP+HDBSCAN to guide the estimation of cluster numbers for GMM clustering of PCA features. Model selection criteria AIC^138^ and BIC^139,140^ were also used to validate the number of clusters choices. GMM clustering was repeated over a small range of number of clusters, and segmentation results were combined using a co-occurrence–based majority voting. Additionally, outlier ions inadequately captured by the principal components were detected, and then segmented independently using multi-Otsu *univariate* thresholding, producing complementary spatial segments missed by multivariate clustering.^142^ The final segmentation was assembled by integrating multivariate clusters and univariate threshold-derived regions.

Conventional clustering of mass spectra based on their similarity typically ignores *spatial context*, which can lead to noisy or inconsistent segmentation. Several studies had explicitly incorporated spatial information to improve segmentation. For example, one work applied total variation (TV) based-denoising to ion images following peak picking to reduce the pixel-to-pixel variability (TV encourages piece-wise smoothness of the images) while preserving sharp structural boundaries.^143^ The resulting denoised pixel data were then clustered using a high-dimensional data clustering (HDDC) algorithm to produce a molecular segmentation more spatially coherent than without denoising.

A spatially aware (SA) segmentation strategy was proposed by Alexandrov and Kobarg.^144^ Specifically, each pixel was represented by an augmented feature vector formed by concatenating the spectra of its neighboring pixels, weighted by a Gaussian function of spatial distance. The higher-dimensional features were then projected into a low-dimensional embedding, followed by k-means clustering, producing smoother and more anatomically coherent segmentation maps. In a structure-adaptive (SASA) variant, the neighborhood weights were further modulated by the similarity between pixel spectra and the spatial center of the neighborhood, yielding an adaptive spatial metric that preserves tissue boundaries.^144^ Building on SASA clustering, Bemis et al. introduced the Spatial Shrunken Centroids (SSC) concept for both unsupervised segmentation (and supervised classification) of MSI data.^141^ The method iteratively estimated “shrunken centroids”, i.e., mean spectra for each ROI that retained the most discriminative *m*/*z* features, and assigned each pixel to a region using spatially aware (SA/SASA) distances between the pixel’s spectrum and the corresponding centroids. A discriminant score was then computed using the distances and converted into pixel-wise membership probabilities to assign each pixel to the segment/ROI with the highest probability. This formulation enabled interpretable segmentation with pixel-level uncertainty quantification. Examples of clustering-based segmentation from SA, SASA, and shrunken centroid are shown in Figure 8a.

**Figure 8:**
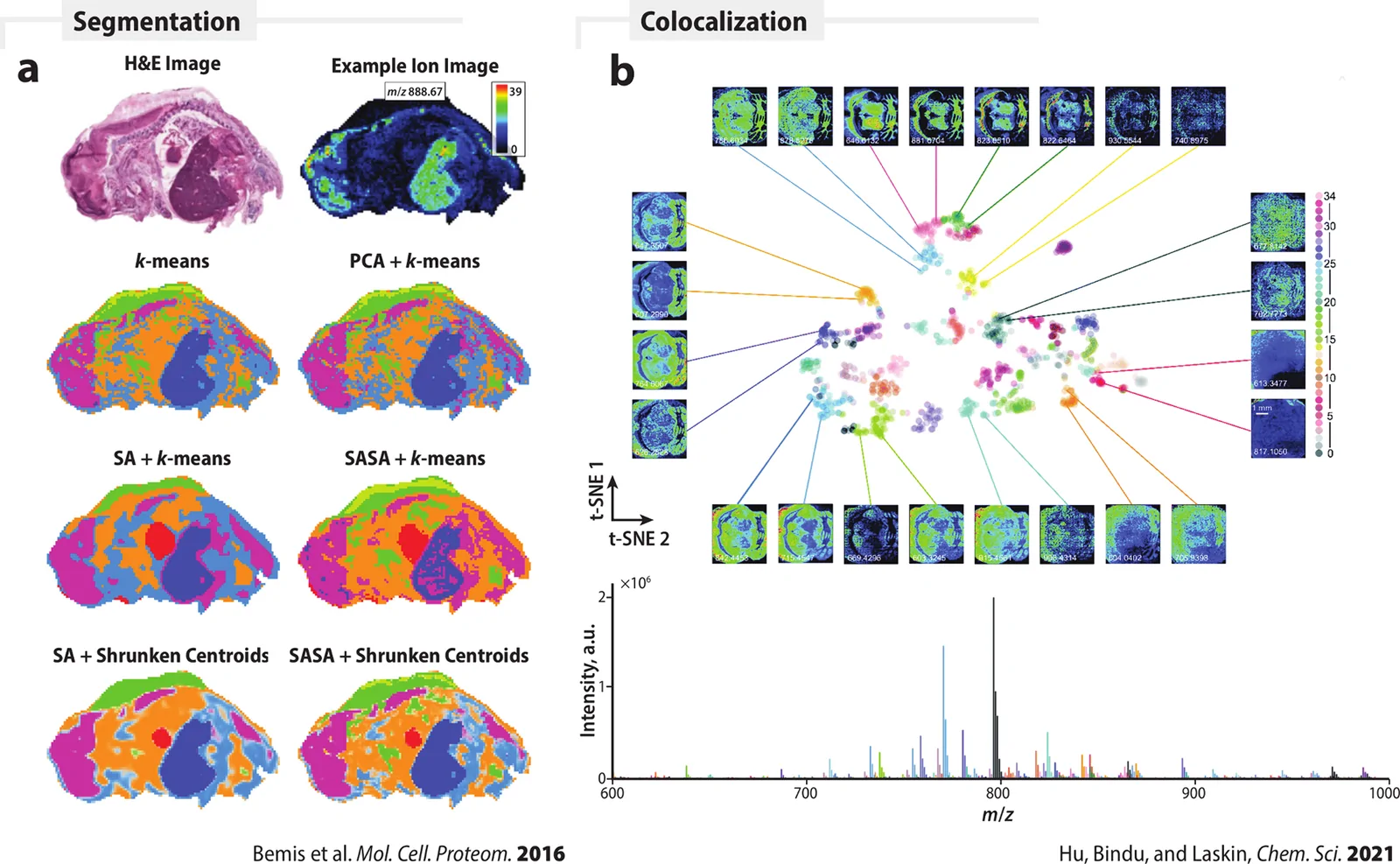
ML-powered segmentation and colocalization. (a) Molecular feature-based tissue segmentation using MSI: An H&E and example ion images are shown, along with segmentation maps from different methods discussed in Section 5.2.1. More anatomically relevant and less noisy segmentation was obtained with methods that used spatial information. Adapted from Bemis et al., 2016. *Mol. Cell. Proteom.* (ref.,^141^ licensed under CC BY 4.0). (b) An example of colocalization using contrastive learning: Each dot in the t-SNE space was from features learned by a contrastive learning method and represents a *m*/*z* ion. Dots close together (ideally) correspond to colocalized ions, with several examples shown. Adapted from Hu et al. 2021 *Chem. Sci.* (ref.,^60^ licensed under CC BY 3.0).

The *clustering-based* segmentation methods discussed above typically rely solely on data-driven structures and do not exploit the anatomical boundaries or tissue morphology that are often known *a priori*. To this end, *semi-supervised* approaches have also been developed. Bruand et al. introduced AMASS which incorporated prior knowledge from tissue morphology through user-defined (or randomly initialized) seed regions as initial clusters.^145^ The algorithm evaluates a **spatial discriminative power** for each *m*/*z* feature to differentiate the initially defined regions, using a rank-based statistic rather than ion intensities (preventing the most abundant ions from dominating the clustering). These statistics were then converted into **log-odds scores**, which represent the likelihood of each pixel belonging to each spatial cluster, and hierarchical clustering of the log-odds profiles iteratively refines the segmentation. By emphasizing internal consistency of molecular patterns rather than strict agreement to anatomical structures, AMASS was able to discover unexpected molecular relationships across different tissue types.

**Deep learning approaches** have become a popular choice for segmentation as they provide more flexible mechanisms to integrate both spectral and spatial information. One such approach used a graph convolutional network (GCN)–based technique that performs unsupervised segmentation followed by semi-supervised fine-tuning using sparse, user-provided scribble annotations to guide MSI tissue segmentation.^146^ A parametric UMAP embedding and GCN-based feature-clustering module were employed. Specifically, each pixel is represented as a node *v*_*i*_, with UMAP embedding derived from its spectrum as the node feature in a graph *G* = (*V*, *E*). A spatial neighborhood graph is constructed by connecting each node only to its immediate spatial neighbors, and edges *e*_*ij*_ are retained when the Euclidean distance between their embeddings falls below a user-defined threshold. The edges encode spectral similarity and spatial context, enabling the GCN to capture local neighborhood structure and tissue topology effectively. Initial segmentation maps produced by the pretrained model are progressively refined through iterative fine-tuning, in which different sets of scribble annotations can be provided at each stage, allowing experts to incorporate domain knowledge and correct ambiguous or biologically implausible regions. In addition, a transfer learning strategy is employed in which models pretrained on a subset of slices are applied to previously unseen MSI data sets, mitigating batch effects across slices and enabling 3D MSI segmentation.

A recent self-supervised MSI segmentation method, called MSInet,^147^ combined pixel-wise UMAP-based dimensionality reduction with a CNN feature extractor and is trained using patch-wise contrastive learning, where nonoverlapping patches exhibiting cosine similarity above a threshold are treated as positive pairs and dissimilar patches as negative pairs. This objective enables the model to learn global semantics without annotations. To further enforce local spatial consistency, a superpixel refinement step groups adjacent pixels with similar textural features and updates pixel labels by majority voting within each superpixel region. MSInet demonstrates improved tissue delineation and robustness to noise compared to several competing methods, including a baseline t-SNE+k-means,^148^ a cardinality-based segmentation,^149^ a GCN-based segmentation,^146^ and a method combining CAE-based feature extraction and seed-based region growing.^128^

#### 5.2.2 Colocalization

While segmentation seeks to group pixels with similar molecular “signatures”, *colocalization* analysis aims to identify molecules that share similar spatial patterns among the thousands of *m*/*z*’s measured in an MSI experiment. Ions may be colocalized due to processes intrinsic to the MSI experiment, such as isotopes or adducts. More interestingly, ions may be colocalized for biological reasons, arising from the same cell type, biological region, or biochemical pathway. As one application, identifying colocalization can discover molecules associated with the same pathology in disease, such as plaque-associated lipids in Alzheimer’s disease or tumor-associated metabolic pathways.

Colocalization can be quantitatively evaluated using a similarity metric for ion images. An early work introduced a correlation-based measure to quantify molecular colocalization, both within a data set and between different MSI experiments.^150^ While suitable for small, relatively simple data sets, pairwise correlation-based approaches can struggle with increasingly large and complex data. One alternative is to formulate colocalization detection as a classification problem, i.e., training a model that takes in pairs of ion images and predicts a “similarity score”. To this end, a semi-supervised learning-based method was developed, leveraging a small set of expert-annotated ion-image pairs as well as a large number of unannotated pairs.^71^ The supervised component ensured the model predictions matched expert-annotated similarity ranks, while the unsupervised component encouraged consistent predictions across augmented views of the same image pair. In this work, the authors also introduced the first open, large-scale gold standard data set for ion colocalization, consisting of 2,210 expert-ranked ion image pairs (with 0 denoting the highest colocalization and 10 the lowest).^71^ Using this benchmark, the authors systematically evaluated a range of classical and learning-based colocalization methods, showing that their own semi-supervised learning method achieved the highest agreement with expert annotations.

Pair-wise evaluation is inherently limited for colocalization detection as multiple molecules (potentially many more than two) can share similar spatial distributions. Dimensionality reduction (Section 5.1.3) can be leveraged for colocalization. Usually, MSI data go through dimensionality reduction in which rows are pixels, and columns are *m*/*z* channels, $X\in R^{P\times C}$ (Figure 3). In this arrangement, each point in the embeddings then corresponds to a pixel. If the input matrix is instead transposed as $X\in R^{C\times P}$ , each point in the embedding corresponds to a *m*/*z* channel, and *m*/*z* channels with similar spatial distributions will cluster in this space. This approach can be further strengthened in combination with self-supervised learning (Section 3.2). For instance, a contrastive learning method was proposed for ion-image colocalization (Figure 8b).^60^ The contrastive module used an ion image and its augmented views (e.g., Gaussian blurring, added noise, and intensity distortion) as positive pairs and different ions as negative pairs, and was able to learn low-dimensional embeddings that form well-resolved clusters of ion images with similar spatial distributions. A linear classifier and spectral clustering were used to generate initial pseudo class labels, which helped enforce consistency by ensuring that different augmented views of the same ion image are assigned to the same cluster. An isotopic recall of 92%, which quantifies the percentage of images of isotopic peaks correctly grouped together, was achieved. Another “noise-robust” ion image clustering method was proposed by Guo et al. that combined image denoising with self-supervised learning.^151^ A two-stage training procedure was employed with a first CAE-based ion image denoising step and a second CNN-based clustering training step.^151^

#### 5.2.3 Classification

In the context of MSI, beyond general spatial tissue segmentation, one might wish to classify pixels as belonging to a given cell type, healthy vs diseased regions, and so on. For example, in a clinical setting, one may wish to decide if a specific tissue region is cancerous. Leveraging pathologist’s annotations as the ground truth, a model can be trained to classify a region of tissue according to its mass spectra. Additionally, assigning pixels to biologically meaningful classes can help improve SNR by averaging spectra within each ROI, enabling more reliable detection of molecules of interest, which in turn can improve classification accuracy.

Several studies demonstrated the efficacy of supervised learning approaches for MSI classification, especially for tumor classification. One early example for tumor classification in MSI is *IsotopeNet*, a CNN architecture adapted to MALDI-TOF with a focus on peptide imaging, and evaluated on two tumor classification tasks: tumor subtyping and primary tumor typing.^152^ Peptide isotopic envelopes are encoded into the CNN’s filter size and stride, thereby better capturing mass spectral structure than a generic CNN. To identify features that drive tissue classification, the authors applied sensitivity analysis, where each *m*/*z* intensity is artificially inflated and the effect on class confidence is measured.

While sensitivity analysis reveals how much class prediction depends on a given *m*/*z* feature, it does not yield a rank-ordered feature importance list. To this end, Tideman et al. proposed a SHAP-based method to interpret the decision-making of an XGBoost classifier trained to classify pixels into user-defined biological regions from their mass spectra.^153^ Specifically, TreeSHAP was used to quantify how each *m*/*z* feature contributed to the classifier’s prediction. Local SHAP scores measure the direction and magnitude of an *m*/*z* feature’s contribution to classifying an individual pixel, while global SHAP scores average this contribution across pixels to rank the most influential *m*/*z* features. The top-ranked *m*/*z* features can be potential biomarker candidates. The authors also introduced SHAP maps, which allow spatial visualization of SHAP scores across the tissue section and provide insight into the spatial specificity of candidate markers.

Annotating every pixel in many “fully supervised” approaches is resource and labor intensive. To this end, methods such as mi-CNN leverage a weakly supervised multiple-instance learning framework that uses a convolutional network to learn from coarse tissue-level labels, avoiding the need for pixel-level annotation in MSI. Although the model is trained only with these weak labels, it was able to infer fine-grained, subregion-level tumor pixels in the ion images. This approach offers a way to bridge the gap between limited annotations and pixel-level diagnostic needs,^72^ and may be explored for tissue/cell classification in large-volume MSI data (high-resolution and/or 3D). A recent work proposed a stability selection-augmented multiple instance learning algorithm (SSAMIL) for TOF-SIMS MSI data to distinguish breast cancer groups according to expression of the protein BMP4.^154^ The method identified discriminating tissue regions from coarse labels, while stability selection-based strategy identified the sparse discriminatory molecular features, enabling interpretation of the classification results and revealing BMP4-associated spatial–molecular signatures.

An alternative direction worth exploring is to leverage multimodal data and annotations generated from a more established modality (e.g., histology stains) to guide MSI classification, potentially combined with significantly reduced human expert supervision for fine-tuning. For instance, a supervised pixel-wise classification method was developed for MSI that operated directly on TIC-normalized spectra *without* requiring peak picking.^155^ The supervised signal is generated by annotating tumor pixels in H&E images adjacent to the MSI section by an expert, which were manually transferred to the MSI data. A variational autoencoder (VAE) with fully connected layers was used to learn a nonlinear latent representation, followed by a classification head for pixel-wise binary tumor classification. Similarly, Race et al. developed a framework for **bidirectional annotation transfer** between H&E images and MSI. Specifically, a deep network model was trained to generate pixel-level annotations from H&E images, which can then be projected onto MSI, enabling histology-based segmentation of MSI. Conversely, they also show that MSI-derived tissue labels/segmentation maps can be transferred back onto the H&E image, allowing MSI-based molecular annotation of H&E.^156^

When the complementary modality is also multichannel, one can use clusters derived in that modality as the supervisory signal. Esselman et al. integrated MALDI MSI with multiplexed immunofluorescence (MxIF) microscopy. Glomerular substructures were first segmented by clustering antibody fluorescence intensities. An ensemble of 10 XGBoost models was then trained to classify MSI data into fluorescence-signature-defined clusters to identify lipids that differentiate glomerular cell types and substructures (using SHAP).^157^ Note that the goals in these developments are not necessarily “matching” the reference segmentation from other modalities, but to discover differentiating molecular features for biochemical insights.

#### 5.2.4 ML/AI-Powered Denoising

Denoising is one of the most studied problems in signal and image processing, though the application of advanced learning-based denoising to MSI has only recently been supercharged. Spatio-spectral denoising in MSI has the potential to improve preprocessing as well as high-level analysis such as segmentation but must account for the signal and noise characteristics unique to MSI data. A few exciting developments have recently emerged. Seydoux et al. developed a denoising strategy that combined PCA and a self-supervised image denoising approach called Noise2Void.^158^ Noise2Void trains a network (e.g., CNN-based) to interpolate artificially and randomly masked out pixels using *noisy images only*. The trained interpolation network can then perform denoising when input with the full noisy images. This approach has found applications in various imaging modalities^159^ and its theoretical justification has been established.^160^ In the work by Seydoux et al., PCA was first used to decompose the unfolded MSI data cube (**X**), and Noise2Void was performed on the score images, then subsequently reconstructing the MSI data cube. An SNR enhancement equivalent to a 16 × 16 spatial binning was achieved with minimal spatial resolution reduction.

While acquiring both high-SNR or clean ion images and noisy counterparts is challenging in practice, there are ways to create “pseudo labels” for training denoising networks from noisy data only, leveraging prior knowledge of mass spectrometry data characteristics. For example, Guo et al. creatively crafted a strategy to pair images of isotopic ions (input) and their monoisotopic counterparts (labels/targets) for supervised training of a U-Net-like denoising network.^161^ The trained network was then used to process all the ion images, demonstrating clear SNR improvements over traditional Gaussian or wavelet-based smoothing without noticeable loss in spatial details. This is similar in spirit to the Noise2Noise approach^162^ for image denoising. As the field grows, we expect to see more advanced MSI denoising strategies. Comparisons of different learning-based denoising methods and evaluation metrics need to be more carefully considered.

### 5.3 Discussion

A range of specific steps in the MSI data analysis chain, from preprocessing to higher-level analysis were discussed in this section. In many applications of MSI, these steps can be creatively combined (and often further innovated upon) to address a particular biological question. Earlier, we discussed how dimensionality reduction approaches can generate informative and efficient representations of high-dimensional MSI data, enabling intuitive visualization using measures such as spatially mapped t-SNE.^122^ Abdelmoula et al. extended this approach, by applying spatially mapped t-SNE to large sample cohorts of gastric and breast cancer tissues.^148^ Edge detection and clustering were performed on the t-SNE representations of the MSI data, and the correct number of clusters was determined by comparing how well the clusters matched to edges in the MSI data. Interestingly, different clusters corresponded to statistically different patient outcomes. This study showed how advanced MSI data analysis can lead to new biological insight.

We recently developed a computational pipeline combining UMAP-based dimensionality reduction and clustering to distinguish 11 anatomical regions of the rat brain based on their lipid profiles.^19^ Using dictionary learning, we identified basis elements of lipid profiles measured for clusters of single cells and mapped them onto a 3D tissue imaging volume, obtaining single-cell level detail without requiring cellular resolution imaging. More recently, we identified rich plaque- and region-specific lipid heterogeneities in Alzheimer’s model mice,^163^ combining plaque-containing pixels masked from fluorescence microscopy and MSI data to reveal region-specific lipid profiles following t-SNE and DBSCAN-based clustering. Using a random forest model, we confidently identified which brain region a particular “plaque-pixel” was in, demonstrating that beyond baseline regional lipid differences, plaques accumulate unique profiles of lipids across the brain. As in just a few other exciting examples, computational MSI workflows are being developed for end-to-end analysis of multimodal fluorescence/MSI data^164^ and for creating organ-wide lipid atlases with minimal technical variation.^165^

A growing body of work attempts to use MSI data with reduced or minimal preprocessing, allowing ML tools to learn patterns in the data while also accounting for variations in the data (e.g., baseline drift, batch effect, and *m*/*z* misalignment etc.) that were conventionally accounted for in a series of preprocessing steps (Section 5.1.1). Abdelmoula et al. developed a “peak learning” strategy by using a variational autoencoder that learned low-dimensional embeddings of MSI spectra with minimal preprocessing, reducing potential bias introduced by user-supervised steps.^113^ Simple peak picking was still applied (local maxima selection) to reduce the data dimensionality before input into the VAE. These encoded features were then used as the input to clustering via Gaussian mixture models, with clusters clearly delineating (for instance) a tumor region in strong agreement with histologically annotated tissue.

A more complete “end-to-end” ML-driven pipeline was developed for LCMS by Deng et al.,^166^ though the principle could be extended to MSI. The only preprocessing applied to the data was resampling the data onto a fixed retention time × *m*/*z* grid to format the data into a tensor the network can read. The model, consisting of multiple CNNs, was applied to identify metabolomic profiles from LCMS of serum samples from lung adenocarcinoma patients vs benign lung nodules and healthy controls. Then, data was input into an ensemble model consisting of a prepooling module, feature extraction, and a softmax classifier. The model outputs classification for each sample, as well as heatmaps and metabolic networks of analytes that drove the classification (generated *post hoc*). It was demonstrated that the ensemble method effectively addressed batch effects by visualizing the flow of the data through forward propagation, highlighting that samples which clustered by batch in the input layer were well separated by sample group in the fifth convolutional layer. While this study did not use MSI, the completeness of the pipeline and its success in classification makes this work worthy of mention, as we envision comparable pipelines will be purpose-built for MSI data sets.

## 6 Computational Multimodal Integration

While MSI alone provides unparalleled spatially resolved, multiplexed molecular readouts, it has intrinsic limits and trade-offs in spatial resolution, speed, molecular coverage, and morphological interpretability due to fundamental physical and chemical principles governing the measurement process. Therefore, the integration of MSI with complementary modalities including optical microscopy,^18,52^ electron microscopy,^167,168^ vibrational imaging (Raman, IR)^169,170^ and other MSI modalities^171,172^ – frequently referred to as multimodal fusion–has become increasingly popular to maximize information from a single experiment and each sample. Moreover, information from immunophenotyping measurements is often needed as the “gold standard” to validate molecular annotations and connect biochemical profiles from MSI to specific cell types and/or protein expression.^173–175^ Traditionally, multimodal workflows relied on manual alignment, simple overlays, and subjective and qualitative comparisons between MSI and images from other modalities. While useful, these approaches lack the rigor and reproducibility needed for high-throughput analysis, introduce bias, and do not fully exploit the rich information distributed across modalities.

Recent advances in AI-powered computational fusion methods are enabling integration of multimodal imaging data. These methods frame the multimodal fusion problem via a data-driven approach—leveraging algorithms for spatial registration, feature extraction, and predictive modeling to align heterogeneous signals. They can be used to combine the high molecular specificity of MSI with the fine structural detail of optical microscopy or spectroscopic techniques, enhancing MSI data and/or producing rich spatial-chemical information that cannot be obtained using any single modality alone. They can also be leveraged strategically to enhance the measurement process itself, e.g., using morphological information from an optical modality (such as fluorescence microscopy) to guide the MSI acquisition of specific tissue subregions and individual cells of interest, maximizing efficiency. The span of instrumental and methodological developments in multimodal MSI has been nicely summarized by Neumann et al., with a focus on various modalities that can be combined with MSI.^176^ In this section, we will dive into key methodologies for computational multimodal fusion and their applications.

### 6.1 Cross-Modality Registration

To jointly analyze or integrate imaging data from two or more modalities, images typically need to be spatially aligned such that features from different modalities can be consistently compared and combined. This necessitates **image registration**. Biomedical image registration is a mature field with many established methods. For a detailed review of image registration as applied to MSI, see Balluff et al.^177^ Here, we briefly outline the registration task and then discuss emerging developments in MSI registration as well as their application to more advanced multimodal analysis.

#### 6.1.1 Image Registration: Basic Principles

In a typical image registration task, one needs to find a coordinate transformation *T*(***r***) (***r*** being the 2D/3D spatial coordinates), to align a *moving image*, *I*_mov_(***r***), to a *target* or *reference image*, *I*_tar_(***r***) (Figure 9a). First, one must decide on the type of coordinate transformation to be applied, and a metric/loss function that measures the “goodness” of alignment. Generally, registration is done by iteratively transforming *I*_mov_(***r***) to minimize the cost function using an optimization algorithm. This process continues until convergence criteria are met or until a set number of iterations have been performed. Image transformations can be *rigid body (translation and rotation only)*, *affine (shearing and scaling included)*, or *more general deformable models*. These operations are illustrated in Figure 9a. Both rigid and affine are global, i.e., the same transformation parameters are applied to all the pixels. When registering MSI data to subsequent imaging obtained on the same section, affine or even rigid transformations are often sufficient. For more challenging registration problems such as registration to an external atlas or between in vivo imaging and MSI data, deformable models that include spatially varying transformation parameters can be particularly useful because they can correct local tissue distortions commonly encountered during tissue extraction and sectioning such as local tears and shrinkage.^19,179^ Commonly used deformable models include optical flow, thin-plate spline, and B-spline based models (see Maintz and Viergever^180^ for a detailed review), with the B-spline being the most common one in MSI. Several metrics/loss functions can be chosen from, with mean squared error (MSE) being a common choice when both *I*_mov_(***r***) and *I*_tar_(***r***) share the same image contrast and mutual information a common alternative for cross modality/different contrast registration.^180–182^

**Figure 9:**
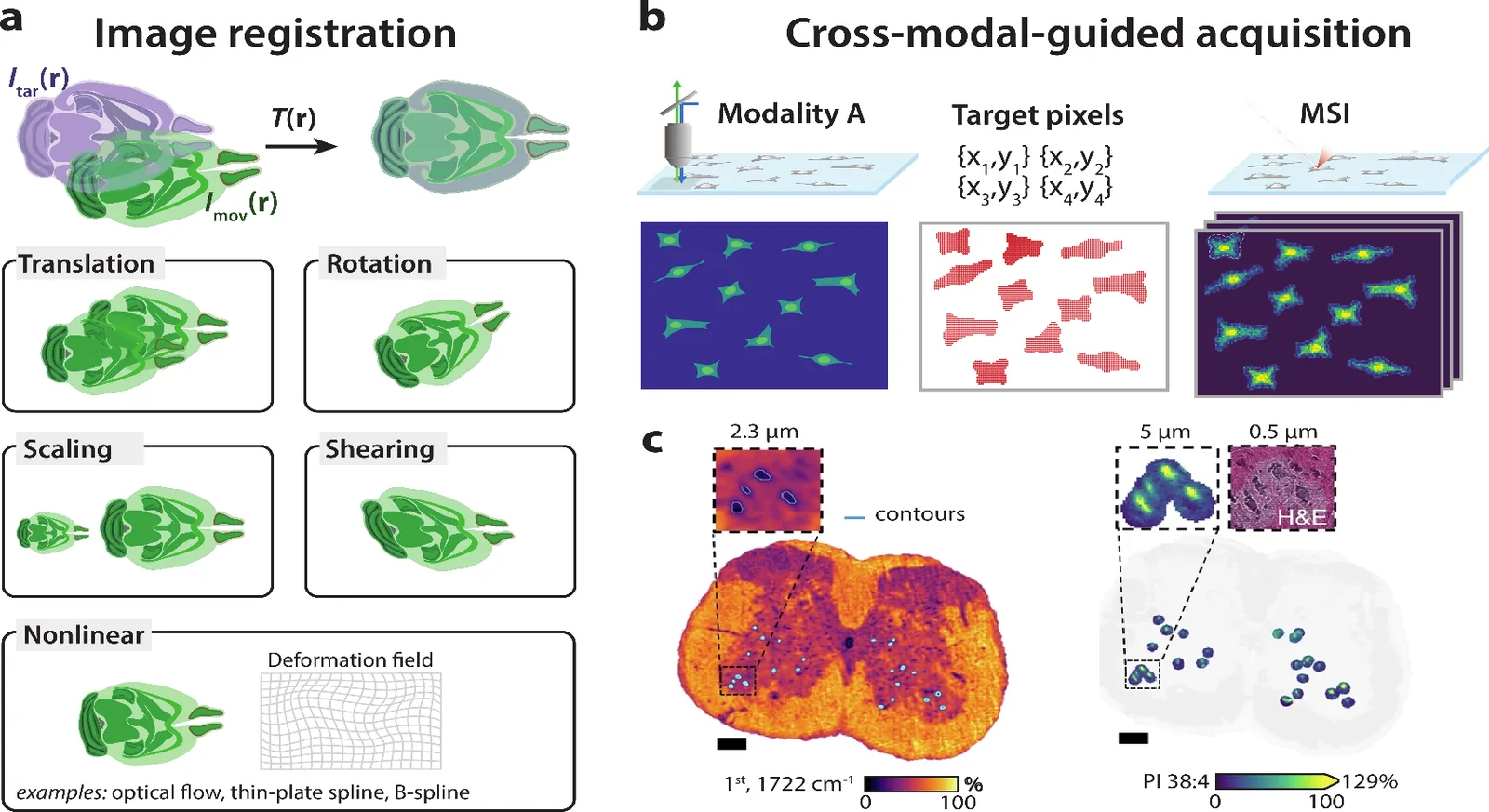
(a) Image registration with different types of transformations considered: Translation and rotation constitute rigid-body registration, while affine registration includes scaling and/or shearing. For the more general nonlinear registration, pixel displacements are not uniform across the image but can be visualized as a deformation field. (b) Cross-modal-guided acquisition: a complementary imaging modality is used to identify regions of interest to target MSI acquisition, increasing effective throughput. An example is shown in (c): An infrared image of a mouse spinal cord (transmittance at 1722 cm^–1^) was acquired in minutes and used to target motor neurons for high-resolution MALDI MSI. Adapted from Gruber et al., *Nat. Commun.* 2025 (ref,^178^ licensed under CC BY 4.0).

Because MSI data is multichannel (hyperspectral), one needs to select either a representative ion image or generate some form of combined image for registration. Afterward, registration can generally be done using existing algorithms. Dimensionality reduction (Section 5.1.3 **)** is particularly useful here; for instance, the first three score images from PCA can be used to generate a pseudo-RGB image for registration, which usually contains richer morphological details than any individual ion image and has a higher SNR. Abdelmoula et al. developed a pipeline using t-SNE to generate a representation of the high-dimensional MSI data with clearly distinguishable anatomical regions, which was then used for registration with paired histological images.^183^ Other approaches have been proposed that leverage information generated during MSI acquisition rather than matching image intensities. For instance, Patterson et al. linked the laser ablation pattern visible in post-MSI microscopy following MALDI imaging to the specific pixel in the corresponding MSI data.^184^ To accomplish this, post-MSI microscopy showing both the laser ablation pattern and tissue morphology is registered to pre-MSI microscopy, using tissue morphology or fiducials visible in both modalities. The ablation pattern is then projected onto MSI pixel grids, so that each MSI pixel can be assigned to its precise location in the pre-MSI microscopy image. This approach can provide more accurate alignment than using MSI data alone, as the registration is done between two microscopy images of higher spatial resolution. Similar registration schemes using ablation craters have been used to map MSI data to microscopy for cultured single cells with high precision^185^ and to align MSI data to spatial transcriptomics from the same tissue section.^186^

#### 6.1.2 Volumetric Reconstruction

Beyond aligning images for joint multimodal analysis, another key application of registration is to align serial sections for faithful volumetric reconstruction.^19,187^ Volumetric MSI captures rich heterogeneities in all three spatial dimensions, provides greater anatomical coverage, and reduces bias from sampling complex structures using only sparse 2D planes.^19,187–189^ However, simply registering serial sections to one another is not sufficient to accurately reconstruct organs with complex 3D geometry. This has been coined as the “banana-into-cylinder” problem, because slices from a curved object (the “banana”) can be perfectly aligned to one another, and yet erroneously straightened into a cylindrical volume.^190^

One approach to address the “banana-into-cylinder” challenge is to register all sections with respect to a reference obtainable from an intrinsically 3D modality, such as MRI (obtained either in vivo or ex vivo). The geometry of this 3D reference constrains each section to its original 3D position. However, there is a practical problem: where does one place each MALDI section along the 3D MRI volume? Or in other words, which “section” from an MRI volume should one pick as the reference for each MSI section, considering that MSI and MRI sections can have rather different orientations and thicknesses? An early work addressing these challenges was presented by Sinha et al. for 3D molecular reconstruction and visualization.^191^ Specifically, blockface images (taking photos of the exposed tissue block between successive cryosections) were taken during sectioning. These images were then stacked into a blockface volume, which acts as an intermediate coordinate system between the MALDI-MSI and MRI data. With this setup, each MALDI image is associated with the corresponding blockface image. The parameters derived from 3D registration between the blockface and MRI volumes were then applied to the MALDI volume, aligning it to MRI for joint visualization and analysis. In their work, only rigid-body transformation was considered to align MALDI to blockface and MRI images. However, in practice, the sectioning process introduces local shrinkage, tears, and other deformations that cannot be adequately addressed by rigid or affine transformation.

Abdelmoula et al. developed a nonlinear MSI to MRI registration pipeline,^179^ an extension of their previous work using t-SNE to generate anatomically informed representations of the MSI data to facilitate spatial correspondence with MRI data.^183^ Specifically, a cubic B-spline deformation model was used,^192^ which achieved superior performance over rigid registration. Similarly, Xie et al. used parametric UMAP to generate representations of MSI data, followed by a combined affine and B-spline registration of 3D sections to a 3D MRI reference.^19^ In both works, the matching MSI and MRI sections for registration were selected manually. Automating the selection of the MRI/reference section remains an important technical issue to address for more efficient and reproducible workflows.

#### 6.1.3 Registration to Atlases

Registration can also be used to align MSI data to existing atlases, enabling automatic extraction of anatomically defined regions and quantitative comparison of regional molecular compositions within a sample, or across large cohorts and different experimental groups. Further, MSI data can be jointly analyzed with the stained markers and/or transcriptomic information available in these atlases to mine for deeper “multiomic” insights. One atlas frequently used with MSI data from the brain is the Allen Brain Atlas (ABA),^193^ which contains coronal and sagittal atlases of the developing and adult mouse brain, the developing and adult human brain, and the mouse spinal cord. Each atlas has detailed anatomical annotations, histological images, and gene expression data, among other information. A catalog of other useful atlases from various species is contained in the Scalable Brain Atlas.^194^

One challenge in registering MSI data to an atlas is selecting the appropriate target image from the atlas that is closest to the stereotaxic coordinates of the MSI data. Toward this end, Abdelmoula et al. developed a strategy for automatic selection of a target atlas image.^195^ First, matrix was removed from sections analyzed by MSI and Nissl stained. They used the hippocampal chord length (the longest straight-line distance across the hippocampus) to select three candidate target images. Then, affine registration was performed and the best atlas target was selected based on the lowest sum-of-squares error calculated between the transformed MSI-matched stain and candidate images. A final B-spline registration was done to refine the registration to the final selected target. Some user intervention is needed, e.g., to calculate the hippocampal chord length, and the workflow does not directly extend to sections without hippocampal anatomy. Moreover, individual MSI sections may not necessarily match any section directly picked from the atlas, e.g., due to angle differences during sectioning. Recently, deep learning tools have been developed to automatically align histological images to atlases. For instance, DeepSlice trained a neural network to predict the coordinates of three corners of an input image w.r.t. the Allen common coordinate framework (CCF),^196^ accounting for the cutting angle, position and orientation of a section and enabling the selection of the most matching “virtual section” from the atlas.

After registration to the atlas, anatomical regions can be automatically segmented from the MSI data to compare region-specific alterations. For instance, Škrášková performed SIMS imaging on *Mfp2* knockout mice in order to determine what metabolite(s) are responsible for the pathologies observed in these animals, and where those metabolites are located.^197^ They performed Nissl staining on sections after SIMS, followed by affine and B-spline registration to the ABA, based on the method developed by Abdelmoula et al.^195^ This workflow revealed the accumulation of long-chain fatty acids in specific brain regions, such as the fourth ventricle, that were not discernible by manual inspection alone. Verbeeck et al. used Nissl-stained images from adjacent sections to match MSI data to the ABA with a similar combined rigid and B-spline registration.^198^ The authors sought to identify what ions were specific to a given region, and what regions contain a given ion. To accomplish this, they built a linear model where each ion was weighted by its presence in a given region. For each ion, this yields an anatomical interpretation of its distribution, e.g., mostly hippocampal, partially cortical, and so on.

Together, these works illustrate the utility of registration to map MSI data to histology, to generate coherent volumes, and to extract anatomically relevant information by mapping to an atlas. In experiments where only registering images obtained from the same tissue section is needed, simple rigid or affine transformations suffice.^183^ However, for aligning successive sections for 3D imaging or mapping to an atlas, nonlinear registration has become a common choice, most often B-spline based.^179,195,197,199^ Going forward, we anticipate that automated, deep learning-based registration tools will become more prevalent, especially with automatic target selection from an atlas.

### 6.2 Cross-Modality-Guided Acquisition

Complementary imaging modalities can be used to improve the efficiency and precision of MSI data acquisition, especially for high-resolution and/or single-cell imaging experiments. Several workflows have been developed to leverage a complementary modality to guide MSI acquisition to target only the tissue regions or cells of interest, minimizing time spent on uninformative regions and enhancing effective throughput. In a few cases, cross-modality information can also help pinpoint specific regions for longer, more informative acquisition modes, such as tandem MS imaging or imaging with ultrahigh mass resolution.

Reducing MSI acquisition time is particularly advantageous when analyzing samples from large clinical cohorts. Heijs and co-workers identified histologically comparable regions on H&E stained myeloid liposarcoma tissue sections.^200^ The annotated sections were then registered to an adjacent section for MSI. Then, only those regions were imaged using MALDI-FTICR MSI for detailed molecular profiling. Recently, autofluorescence and brightfield images taken from *the same section*, *prior to MSI*, have been used for guided acquisition.^201^ Regions annotated manually or determined by an algorithm were used to select ROIs. Analyzing the ablation craters (for MALDI methods) helps confirm that the correct areas were targeted, and additional immunofluorescence can be performed post-MSI.^184^ Most recently, mid-infrared (MIR) imaging using quantum cascade lasers (QCLs) has been used to guide MSI acquisition.^178^ Specifically, whole tissue sections were imaged in just minutes by QCL-based IR with a 5 × 5 μm^2^ pixel size, identifying characteristic stretches for lipids, proteins, and nucleic acids with fine morphological details.^202,203^ The authors then used select IR features to target ROIs for MALDI imaging (Figure 9c). Using several lipid-correlated IR features, the authors used targeted MALDI-TIMS-MS^2^ a highly information-rich MSI mode that is rarely applied for large tissue sections due to approximately 10-fold longer run times than standard MSI.^204,205^ Using selective IR stretches for guidance, the authors characterized over 100 sulfatide species in a mouse model of sulfatide enrichment.

The analysis of dispersed or cultured cells is another excellent use case for guided MSI, as eliminating the time spent imaging regions between individual cells greatly increases information content per unit time. With the open-source package microMS,^206^ the locations of cells (or other dispersed objects) on a slide can be first identified by microscopy and object detection, often aided by MSI-compatible stains like Hoechst. Then, using a graphical user interface (GUI), the user then maps cell locations on the microscopy image to their physical coordinates on the instrument stage using the in-source camera equipped in many commercial mass spectrometers. The stage is then directed to only those regions. This approach enabled tens to hundreds of thousands of cells to be analyzed within one experiment.^207,208^ Strictly speaking, this is a *one-measurement-per-cell* approach. A similar microscopy-guided approach, termed FISCAS, performed targeted *imaging* of single cells.^209^ FISCAS uses Bruker’s commercial software flexImaging to perform registration between pre-MSI microscopy and MSI stage coordinates. As this is less accurate than the registration approach of microMS, a “safety margin” of 50 μm was expanded around detected cells. Microscopy data can be further integrated into the data analysis. As proof-of-concept, the authors demonstrated a correlation between the intensity of a lipid droplet stain and the triacylglycerol MS signal across a thousand assayed cells. Another notable method, SpaceM, used microscopy details *post hoc* to attribute measured metabolites and lipids to particular cells.^185^ To this end, MALDI laser ablation patterns measured post-MSI are coregistered with pre-MSI microscopy. The spectrum associated with a particular ablation mark can then be associated with the cell(s) that were sampled. Compared to microMS or FISCAS, SpaceM is designed for cells cultured at high confluence, as large regions are imaged completely (containing thousands of cells) and cell-specific details are teased apart via analysis rather than strictly guiding the MS acquisition. The same group later released HT-SpaceM, an extension with improved detection of small molecule metabolites, on-slide wells generated by laser etching, and quality control assessments.^210^

### 6.3 Multimodal Fusion for Spatial and Chemical Enhancement

#### 6.3.1 Cross-Modality Guided Analysis

Besides guiding the acquisition, modalities that provide more morphological details than MSI and/or complementary information can also enhance data analysis. For example, electron microscopy (EM) provides nanometer-scale resolution and can be used to identify ROIs at subcellular levels. Du Toit et al. developed a method using the object detection framework YOLO (you only look once) from computer vision for segmenting ROIs in EM images (e.g., multiple organelles) for registration with images from NanoSIMS and extracting chemical information from individual subcellular ROIs.^167^ Jones et al. used fluorescence emission microscopy with a genetically encoded fluorophore to identify specific ROIs (splenic germinal centers) on tissue sections, and to achieve high-accuracy registration between multiple modalities for joint analysis.^211^ The workflow integrated multiple modalities (fluorescence, H&E, and MSI) into a unified multiplanar data set, enabling cross-modal analysis that offers a unique combination of spatial resolution, molecular coverage and cell-type and activity state specificity. A multivariate linear model based fusion of coregistered multimodal images was used,^18^ revealing correlations between fluorophore expression and ion distributions, and uncovering 16 germinal-center-enriched lipid species.^211^ Acquiring both MSI and immunohistochemistry (IHC) data from the same tissue section can also help validate molecular annotations, and derive cell-type-specific biomolecular profiles from MSI, with IHC serving as a gold standard for cell typing.^174,175,212,213^

#### 6.3.2 Image Fusion to Enhance MSI

As discussed in earlier sections, while offering unparalleled chemical details, MSI has a much lower spatial resolution than optical and electron microscopy modalities. This makes their integration to combine high spatial resolution and rich chemical detail appealing (Figure 10a). As one of the first examples, Van de Plas et al. proposed a multivariate regression method to integrate H&E stains with MSI data for enhancing the resolution of chemical images.^18^ The relationships between patches of MSI and H&E images were learned in the latent spaces at lower-resolution grids and used for inference at higher-resolution grids, including prediction of ion distributions in regions where MSI measurements are missing. A resolution enhancement of up to 10-fold or more for MALDI MSI was reported. This fusion approach was later used in various multimodal imaging studies. For example, data from fluorescence microscopy and the “single-probe” MSI technique (an ambient surface-sampling technique similar to nano-DESI)^214^ were fused to super-resolve the MSI images from 17 to 5 μm and identify altered metabolomic features associated with amyloid plaques.^215^ In a different study, H&E stain images were fused with ambient ionization MSI modalities such as DESI and nano-DESI using the multivariate regression algorithm to achieve higher resolution biomolecular imaging, enabling biomarker discovery from mouse metastatic lung and human breast cancer tissues.^216^ A similar fusion strategy was also applied in Jones et al. to mine correlations between fluorophore expression and ion distributions (discussed above).^211^

**Figure 10:**
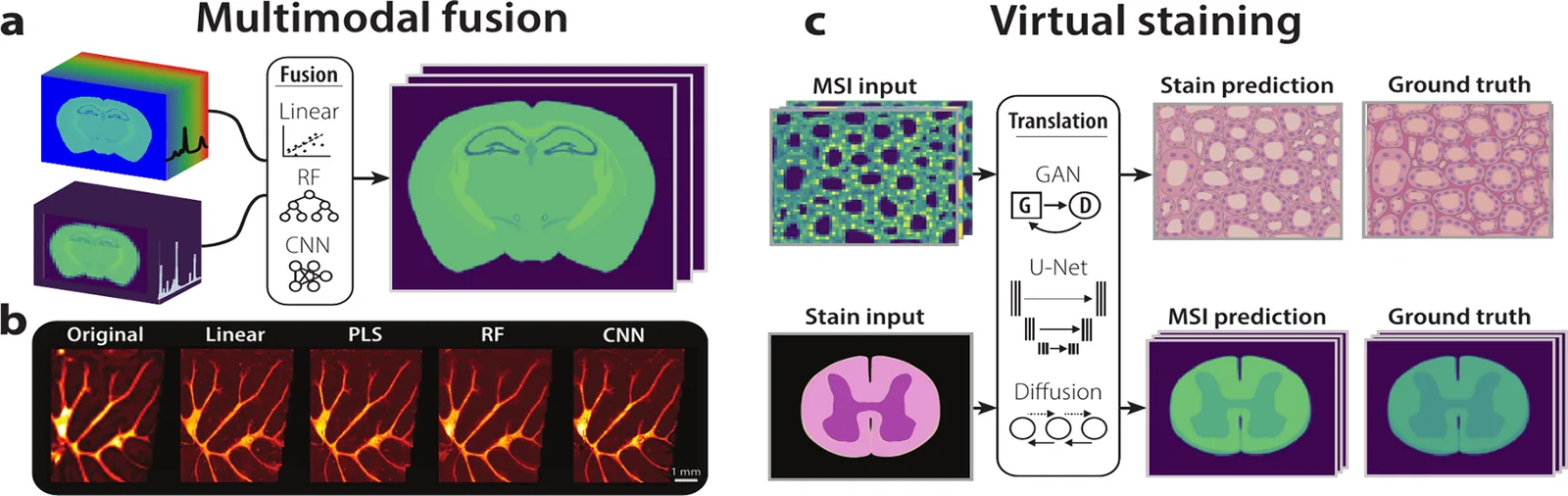
Multimodal fusion: (a) MSI data can be integrated with a complementary modality via computational means to enhance data quality and information content. (b) An example of fusing MSI and H&E microscopy to achieve super-resolution of MSI; Adapted with permission from Liang et al., *J. Am. Soc. Mass Spectrom.* (ref,^219^ copyright 2024 American Chemical Society). (c) An illustration of the virtual staining concept: MSI to stained images prediction (top), or stained images to MSI prediction (bottom). Different deep network-based image translation models (and combinations thereof) can be used, including GANs, U-Nets, and diffusion models (see Section 3 for generative modeling).

Ščupáková et al. developed a patch-based super-resolution fusion algorithm that used high spatial-resolution features from one modality (optical microscopy with histology stains) to guide the reconstruction of an image from a low-resolution modality (MSI).^87^ For the reconstruction of each pixel at the high-resolution grid, the algorithm estimates the ion intensity from the neighboring pixels guided by both tissue segmentation and the intensities of the high-resolution histology image, then iteratively enforces consistency with the original low-resolution measurements, producing spatially sharpened MSI images aligned with histological features.

As one of the first attempts to combine IR spectroscopic imaging (QCL) and MSI, Neumann et al. reported a method to sharpen MALDI-MSI data using a subsequent IR acquisition with a higher resolution on the same section.^24^ Specifically, a pan-sharpening strategy using the Laplacian pyramid method was employed, which decomposes images into multiple spatial frequency layers. The high-frequency information from the IR images was then used to sharpen the MSI data, enhancing spatial detail while preserving chemical integrity. To enhance the spatial delineation of segmentation maps derived from MSI data, Alexandrov et al. proposed a super-resolution method based on morphological operations.^1001^ This method combined upsampling of the segmentation map, applying morphological erosion to introduce boundaries between regions, refining these boundaries per region, and finally morphological dilation to reconstruct smooth, higher-resolution segmentations without eliminating or splitting any regions. These maps can then be overlaid onto microscopy images for more precise histological interpretation.

An interesting example of cross-modality fusion involves combining MALDI and SIMS. MALDI offers relatively soft ionization with minimal fragmentation but limited spatial resolution, whereas SIMS provides submicrometer resolution at the expense of extensive molecular fragmentation—making the two modalities highly complementary. Assuming a linear relationship between MALDI and SIMS ion signals, Borodinov et al. applied NMF to each data set to obtain corresponding loading maps (after registration and grid matching). Canonical correlation analysis (CCA) was subsequently used to identify cross-modal correlations between the NMF loadings. The resulting transformation matrix enabled reconstruction of one modality from the other, e.g., generating MALDI-like spectra at SIMS spatial resolution.^217^ Using a similar fusion strategy to correlate multiple imaging modalities – MALDI, SIMS, Raman and H&E – Race et al. demonstrated substantial gains in MSI performance including spatial resolution enhancement (by transferring fine spatial features in SIMS to MALDI and Raman), out-of-distribution prediction (e.g., using H&E to predict MALDI ion distributions), and effective removal of modality-specific artifacts.^218^

Deep neural networks, when appropriately designed and trained, can effectively extract the multiscale features from different modalities and mine their nonlinear relationship for improved fusion, addressing the limitations of “conventional” methods that require hand-crafted or manually selected features and linear assumptions. One active area of pursuit is leveraging a complementary modality to achieve super-resolution of MSI. This is attractive as it is difficult to apply generic DL-based methods for image super-resolution in MSI, because acquiring paired low- and high-resolution MSI data sets from the same tissue section for training super-resolution models is quite challenging. It is possible to simulate low-resolution ion images from high-resolution data. However, it requires an accurate model of the acquisition process, which can be difficult due to laser design/configuration constraints on the systems. Moreover, the highest spatial resolution achievable in MSI is still limited by the laser spot size and sample preparation (i.e., analyte delocalization due to tissue washes and matrix application) and modalities such as optical microscopy do offer strong complementary information at higher resolutions, e.g., morphological priors.

One approach aims to fuse MSI with higher-resolution reference images (e.g., from optical microscopy) to enhance resolution.^88^ Similar ideas have been explored (discussed above) but this time using networks such as CNNs to learn the relationship between the higher-resolution structural priors (from microscopy) and lower-resolution MSI data. Liang et al. developed a fusion method that integrates MSI with stained fluorescence and brightfield microscopy to study the distribution of pharmaceutical compounds.^219^ They experimented with a number of models, including linear regression, random forest, and CNNs to predict higher-resolution MSI data based on a microscopy input. CNN was shown to offer superior performance and higher accuracy (Figure 10b). While these approaches are exciting and generate mesmerizing images with rich molecular information, we encourage the community to be cautious about the potential bias introduced and interpretation of the results, especially considering super-resolution is an inherently ill-posed problem. Model-based data consistency enforcement and/or other mechanisms may be considered^90^ to help ensure fidelity and biological accuracy.

#### 6.3.3 Virtual Staining

MSI experiments are often contextualized with histological staining, either on the same section or a serial section. Staining provides a clear anatomical reference, enables interpretation and annotation by histologists, and facilitates the use of a number of atlas-guided workflows that have been developed for stained images. Virtual staining – generating stain-like images from label-free ones – represents a new paradigm in microscopy imaging (Figure 10c).^220,221^ With MSI’s label-free nature and rich molecular profiles generated at each pixel, it is reasonable to expect that it can be used to generate stain-like contrast on the same tissue section. Zhang et al. developed a generative AI–based method for virtual staining from MSI.^25^ The method used a conditional diffusion image model, in which low-resolution ion images serve as the conditioning input to a noise estimating U-Net to generate high-resolution “pseudo”-histological images (10× higher resolution). In contrast to cross-modality super-resolution approaches discussed earlier, this method did not rely on an additional modality such as H&E or IHC on the same section for image synthesis during inference, thus eliminating the need for chemical staining and cross-section/modality registration while still capable of producing morphological information. While not advocating for replacing staining with MSI, we note that such a *coregistered* virtual stain image can be useful for MSI data analysis, because it allows us to leverage the many more established tools available for histological images, e.g., registration and segmentation, in the overall workflow.

As MSI acquisition is expensive and time-consuming, another line of work explores the generation of molecular information directly from histological images such as H&E. Ul Haque et al. developed a method that uses paired H&E and MSI images to train a model to predict molecular information directly from H&E, enabling chemically interpretable cancerous regions to be identified in H&E images.^222^ In this work, the author trained a linear regression model to predict PCA components of MSI from features extracted from H&E images (using a pretrained ResNet-50 network), demonstrating “virtual MSI”. By predicting the PCA components instead of raw spectra or complete *m*/*z* features, the method drastically reduced the dimensionality of the problem while preserving 98.51% of the variance of the data. The predicted MSI components are then used with a logistic regression to classify cancerous regions.^222^

## 7 Future Directions

As advanced ML/AI methods continue to infiltrate every step in the MSI workflow and transform how data are acquired and analyzed, we are witnessing accelerating convergence of analytical and computational approaches, resulting in new methodologies and tools to automate workflows, improve throughput, enhance resolution, and extract more reproducible chemical and biological information. Meanwhile, there is significant room for continued innovation, and many aspects remain under-explored. In this last section, we highlight a few possibilities and trends that will shape the field of computational MSI.

### 7.1 Advanced Instrumentation Powered by AI

As the portfolio of MSI instruments continues to grow and evolve, there is tremendous potential or perhaps even a necessity for ML/AI tools to work in synergy with advanced equipment. Many new hardware and detection approaches are emerging. One example is the combination of trapped ion mobility (TIMS) and FTICR in Bruker’s timsMRMS system.^223,224^ Equipped with a MALDI source, these instruments can be used for imaging which will provide data at unprecedented scales while facing a major throughput bottleneck. Because each FTICR acquisition event is on the order of seconds, while TIMS separation is on the order of hundreds of milliseconds, these instruments are operated in gated mode, where only a selected window of TIMS-separated ions enter the FTICR cell, with the window sequentially stepped until the desired TIMS spectrum is obtained. This lengthy process may be accelerated using the approaches discussed in this review (Sections 4.1 and 4.2) to make imaging experiments more practical. There will also be significant memory and computational challenges to handle and analyze these high-dimensional data. For instance, a single 2-million-point FTICR transient is megabytes of data, and the size of raw data in a hypothetical MALDI TIMS FTICR experiment could be several hundred folds bigger, thus hundreds of MBs for just a single pixel! Of course, the actual file size depends on the data formatting, binarization, and compression strategies. Nonetheless, it is clear that efficient data representation and processing strategies are needed for these high dimensional data types. Alternatively, cross-modality guided acquisition (Section 6) could be used to guide TIMS-FTICR or other higher-dimensional scans to only selected pixels of interest. Such approaches have been applied to ultrahigh mass resolution 21 T FTICR instruments.^225,226^ Several other ion mobility MSI approaches are appearing and each of these has a range of performance specifications enabling enhanced data but also a separate set of challenges.^46,227,228^

While current MSI toolkits are largely applied to lipids and small metabolites, several approaches have recently emerged for imaging proteoforms^28,38,227^ and resolving higher-order complexes^229^ by coupling nano-DESI or DESI with high-resolution MS. Spatially resolved proteoform imaging can resolve a vast functional landscape of proteins that are invisible to transcriptomics, such as post-translationally modified species, those arising from RNA splice modifications, polymorphisms, and higher-order complexes.^230,231^ Proteoform imaging mass spectrometry (PiMS) combines nano-DESI with Orbitrap-based charge detection mass spectrometry.^28,232^ While imaging proteins by MSI is not new, previous work was limited to select abundant species, and higher-order complexes were not observed. These recent developments are pushing MSI toward true spatial proteomics, though the spatial resolution is relatively poor and the number of proteins detected still lags far behind LCMS. Advanced denoising, super-resolution, and acquisition, developed in concert with hardware, sample preparation and detection strategies, may be useful for enhancing these techniques and improving molecular characterization.

The rich spatial-chemical detail provided by MSI has historically come at the cost of chemical depth and annotation depth compared to hyphenated techniques like LCMS. While the focus of this review is how ML/AI-powered computational approaches impact imaging performance (the “spatial” aspects), we note that confident molecular annotation is a technical challenge which requires computational solutions. METASPACE, which uses spatial information and a target/decoy approach,^23,233^ has been a widely used resource in the MSI community. On the hardware side, many commercial MSI instruments now include ion mobility, which can provide collision cross sections (CCS) as an additional matching metric.^234^ Approaches for spatially resolved on-tissue tandem MS significantly improve annotation,^204,235,236^ but are limited by the computational pipelines available. New approaches such as single-ion detection^28,232^ create unique challenges for chemical assignments. Realizing the full benefit of these new instrumentation capabilities will need equally creative computational innovations. While MSI might never truly match the chemical depth of LCMS, we anticipate that computational tools will substantially narrow this gap.

It is also intriguing to consider the possibility of more “intelligent” (or adaptive) MSI instrumentation, which combines various agents trained to automate procedures, optimize parameters and adaptively image the sample to maximize performance of a specific task or for a specific hypothesis. Reinforcement learning (Section 3) is a promising direction and has already shown promise for optimizing analytical methods, such as liquid chromatography.^237,238^ In the context of MSI, one “straightforward” application of RL is to design adaptive sampling policies for tissue imaging, where the system can “intelligently” select the most informative next pixels to scan, given the already scanned locations and evaluation of certain performance metrics, and automatically stop when an accuracy threshold is met instead of performing a full raster scan. Related work in other imaging modalities (e.g., X-ray^75^) has demonstrated the feasibility of using RL to control the scanner to adaptively balance between diagnostic accuracy and acquisition time. Furthermore, adaptive MSI acquisition can guide not just where to sample the next pixel, but also in what mode. For instance, RL-guided MSI can determine what length of transient is needed, where to obtain MS/MS or ion mobility information from on-the-fly decision making. RL-enabled “self-optimizing” instrumentation, using RL to determine selecting voltages for ion transfer optics, collision energies, and so forth, is another promising direction that has only recently become a realistic possibility.

### 7.2 Scaling Up Measurements

As MSI instrumentation and computational tools improve, the size and scale of measurements will continue to grow (Figure 1a). Strategies to enable efficient scaling of measurements are needed for imaging at high spatial resolution and in three dimensions. Recently, physically expanding tissue sections in a hydrogel has attracted considerable interest in the MSI field to improve spatial detail.^239–242^ As tissue is expanded, the number of pixels needed to cover the same section increases, effectively lengthening acquisition time and making acceleration methods desirable (Section 4).

While the first reports of 3D MSI of large biomolecules were published 20 years ago,^243^ 3D MSI remains highly specialized and niche. However, thanks to the improved hardware performance and advanced computational approaches discussed in this review (e.g., addressing speed, alignment and segmentation issues), 3D MSI is becoming more practical.^19,187^ The majority of 3D MSI experiments are accomplished by serially sectioning tissue, imaging each slice, and computationally combining each section into a volumetric representation (for a description of other approaches, such as depth profiling, see Palmer and Alexandrov^244^). This scaling up effort to the eventual faithful volumetric reconstruction has several challenges that need to be addressed by ML/AI-powered computational tools. For example, many slices need to be imaged for real 3D imaging (not just a few slices that are far away), especially when targeting organ-wide investigation, which can result in prohibitively long imaging times and constrain sample size. Acceleration in the spatial and/or temporal dimensions (Section 4) can help. One may even consider sparse sampling along the *z*-axis, varying density of sections retained for analysis. The feasibility of this approach is supported by the work of Vos et al., who investigated the fraction of sections from a large 3D imaging experiment of clinical carcinoma samples needed to retain molecular heterogeneity.^245^ It was found that 4 out of 20 sections were sufficient, enabling them to map glycans across a total of 24 clinical specimens, supporting the promise of prospective 3D sparse sampling. However, the degree of sparse sampling that can be tolerated in 3 (or 2) dimensions will depend on the specific sample type, the extent of spatial heterogeneity present and research questions. Image fusion (Section 6) may also play a key role in scaling up the measurements. Multimodal data will not only provide a holistic and systems-wide view of the tissues/cells of interest but also help to impute missing measurements. Virtual staining may relax the need to stain all serial sections in a 3D experiment.

Achieving high-throughput, reproducible 3D MSI will also allow new avenues for *multiscale* mass spectrometry acquisition and analysis. Particularly, joint analysis of data from whole organs (or even whole bodies) and single cells or subcellular structures holds the potential to reveal biochemical organization that is inaccessible at any individual scale. Although it is possible to acquire organ-wide or organism-wide data at subcellular resolution, we do not imagine such an effort can be replicated by most research groups.^246–249^ Multiscale MSI requires new innovative ways to integrate data acquired at different scales. We recently applied dimensionality reduction to MS data from thousands of individual brain cells, learned the low-dimensional dictionaries for each cell cluster, and fit them to 3D tissue MSI data.^19^ This allowed us to visualize the spatially varying cell-specific contributions without having to image the tissue physically at cellular resolution and at a practical throughput. Adding ground-truth cell type information can further enhance this workflow. Computational single cell mass spectrometry is also a rapidly evolving field,^250^ and many of the tools described in this review also apply there, whether the data are one measurement per cell or imaging at single-cell resolutions.

As AI-powered computational workflows are increasingly adopted in various MSI applications, *generalizability* should be carefully evaluated. Most (or a majority of) methods are demonstrated on the rodent brain and kidney, which serve as powerful testbeds due to their rich chemical heterogeneity, clear anatomical organization, ease of sampling and sectioning, and easier benchmarking with the wealth of existing literature on these systems. Assessing method performance and robustness over different sample types, instruments, laboratories and/or operators is important for broad adoption. Expanding the size and diversity of publicly available benchmark data sets would substantially facilitate such efforts, especially for groups that have limited access to certain instruments and sample types. Consensus on data quality control, methodology recommendation, and performance metrics may be needed to sustain these efforts.

### 7.3 Multi-Omic Acquisition and Integration

By producing large-scale, sensitive and specific measurement of biomolecules in their native tissue locations, MSI bridges a major gap in the current “spatial omics” landscape.^251^ Specifically, spatial *metabolomic* or *lipidomic* data obtained by MSI nicely complement the gene expression data produced by the relatively more advanced spatial transcriptomics tools, to generate a more complete portrait of the biological pathways that modulate functional or disease states and their transitions.^252–254^ This field of multiomic imaging, in a stronger sense than mainly combining different forms of spatial transcriptomics, is still at an early stage of development. A significant amount of effort is still focused on addressing the technical challenges for same-section multiomic data acquisition,^186,252,254^ for instance, significantly fewer transcripts are detected in a tissue section after MALDI MSI vs before,^186^ or performing MSI and transcriptomics on separate tissues or cells for joint analysis.^253^ Moreover, as transcripts indirectly produce metabolites and lipids and the pathways may be separated by long spatial distances and times, analysis and integration of such multiomic data require a deep understanding of underlying biological pathways. In the coming years, as methods and technology for multiomic acquisition become more robust and widely adopted, we anticipate that advanced biology-rooted, ML/AI-powered computational strategies and data science tools will become an integrative component of multiomic research, which is producing data with ever-increasing dimensions and complexity beyond human comprehension, if it is not already so.

For single-cell multiomics, several approaches have been reported besides MSI,^255,256^ though lacking the *spatially resolved/imaging* aspect. Several challenges need to be addressed. First, spatial resolution varies substantially between imaging modalities and even within MSI. Analysis order and sample preparation need to be carefully considered and optimized in order to achieve sufficient data from a tissue section that has already been analyzed by a given modality. Finally, the statistical distributions between spatial omics often differ; for instance, spatial transcriptomics yields sparse count data whereas intensity data from MSI is continuous.

As one example of spatial multiomics applied to sequencing-based omics, but with promise for MSI, Long et al. introduced SpatialGlue.^257^ SpatialGlue is a GNN-based model that learns modality-specific embeddings and then fuses features from different modalities via attention to obtain an integrated representation.^257^ With this approach, the authors were able to identify fine tissue spatial domains (e.g., spleen macrophage subtypes) with substantially better accuracy than any unimodal approach. More recently, Liu et al. linked MSI-based spatial metabolomics with gene expression data from an adjacent slice and reported that the integrated data could resolve both anatomical and pathological domains in the mouse brain that were not discernible with either individual technique.^258^ We anticipate that similar strategies will be applied to multiomic studies involving MSI data, ideally using the same section for different modalities. However, approaches to address the discrepancy in spatial resolutions, imaging order, and statistical distributions between MSI and other spatial omics tools are under development.

## 8 Summary

In this review, we examined the interplay of mass spectrometry imaging and artificial intelligence, spanning from MSI acquisition paradigms and learning frameworks to cutting-edge computational innovations in data acquisition, reconstruction, and analysis, with a special emphasis on their impacts on imaging performance. We explored how spatial and temporal acceleration strategies are enhancing imaging throughput, how super-resolution techniques are pushing beyond physical limits, and how advanced dimensionality reduction, clustering and classification approaches are extracting chemical information from increasingly complex, high-dimensional data sets. We also discussed the expanding role of multimodal data fusion in enabling more efficient acquisition and overcoming resolution and information barriers inherent to MSI. With the rapid advancement of AI and computing technologies, computational MSI is poised for accelerated growth, transforming instrumentation design, experimental workflows, and analytical pipelines. These advances call for a new generation of researchers and practitioners who can bridge analytical chemistry and computational tools. We envision that analytical chemists proficient in ML/AI and computational tools, alongside ML/AI researchers well-versed in analytical chemistry and mass spectrometry, will be critical in driving innovation and translating these technologies into practical solutions for biological discovery and clinical applications.

## Abbreviations

ABA: Allen Brain Atlas
AI: Artificial Intelligence
AMASS: Algorithm for MSI Analysis with Semi-supervised Segmentation
BH: Barnes-Hut
CAE: Convolutional Autoencoder
CNN: Convolutional Neural Network
COW: Correlation Optimized Warping
CPU: Central Processing Unit
DBSCAN: Density-based Spatial Clustering of Applications with Noise
DESI: Desorption Electrospray Ionization
DL: Deep Learning
DLADS: Deep Learning Approach for Dynamic Sampling
EM: Electron Microscopy
FDR: False Discovery Rate
FT: Fourier Transform
FTICR: Fourier Transform Ion Cyclotron Resonance
GAN: Generative Adversarial Network
GCN: Graph Convolutional Network
GMM: Gaussian Mixture Model
GNN: Graph Neural Network
GPU: Graphics Processing Unit
HDDC: High Dimensional Data Clustering
H&E: Haemaotoxylin and Eosin
ICA: Independent Component Analysis
IHC: Immunohistochemistry
IM: Ion Mobility
IPCA: Incremental Principal Component Analysis
IR: Infrared
LCMS: Liquid Chromatography Mass Spectrometry
MALDI: Matrix-Assisted Laser Desorption Ionization
ML: Machine Learning
MNF: Minimum Noise Fraction
MOSR: MSI from Optical Super Resolution
MRI: Magnetic Resonance Imaging
MSI: Mass Spectrometry Imaging
nano-DESI: nanospray desorption electrospray ionization
NMF: Non-negative Matrix Factorization
PCA: Principal Component Analysis
PSNR: Peak Signal-to-Noise Ratio
QCL: Quantum Cascade Laser
RAM: Random Access Memory
ReLU: Rectified Linear Unit
RL: Reinforcement Learning
ROI: Region of Interest
SASA: Spatially-Aware Structure-Adaptive
SHAP: SHapley Additive ExPlanations
SIMS: Secondary Ion Mass Spectrometry
SLADS: Supervised Learning Approach for Dynamic Sampling
SSC: Spatial Shrunken Centroids
t-SNE: t-Distributed Stochastic Neighbor Embedding
TIC: Total Ion Count
TIMS: Trapped Ion Mobility
TOF: Time-of-Flight
UMAP: Uniform Manifold Approximation and Projection
VAE: Variational Autoencoder
